# Integrated analysis of the role of PR/SET domain 14 in gastric cancer

**DOI:** 10.1186/s12885-024-12424-1

**Published:** 2024-06-05

**Authors:** Xiao Li, Cong Wang, Youcai Wang, Xiaobing Chen, Zhi Li, Jianwei Wang, Yingjun Liu

**Affiliations:** 1 Affiliated Cancer Hospital of Zhengzhou University, Henan Cancer Hospital, Zhengzhou, China; 2 School of Computer and Artificial Intelligence, Zhengzhou University, Zhengzhou, China

**Keywords:** Gastric cancer, PRDM14, Apoptosis, NANOG, Prognostic model

## Abstract

**Background:**

Gastric cancer is one of the most common tumors worldwide, and most patients are deprived of treatment options when diagnosed at advanced stages. PRDM14 has carcinogenic potential in breast and non-small cell lung cancer. however, its role in gastric cancer has not been elucidated.

**Methods:**

We aimed to elucidate the expression of PRDM14 using pan-cancer analysis. We monitored the expression of PRDM14 in cells and patients using quantitative polymerase chain reaction, western blotting, and immunohistochemistry. We observed that cell phenotypes and regulatory genes were influenced by PRDM14 by silencing PRDM14. We evaluated and validated the value of the PRDM14-derived prognostic model. Finally, we predicted the relationship between PRDM14 and small-molecule drug responses using the Connectivity Map and The Genomics of Drug Sensitivity in Cancer databases.

**Results:**

PRDM14 was significantly overexpressed in gastric cancer, which identified in cell lines and patients’ tissues. Silencing the expression of PRDM14 resulted in apoptosis promotion, cell cycle arrest, and inhibition of the growth and migration of GC cells. Functional analysis revealed that PRDM14 acts in epigenetic regulation and modulates multiple DNA methyltransferases or transcription factors. The PRDM14-derived differentially expressed gene prognostic model was validated to reliably predict the patient prognosis. Nomograms (age, sex, and PRDM14-risk score) were used to quantify the probability of survival. PRDM14 was positively correlated with sensitivity to small-molecule drugs such as TPCA-1, PF-56,227, mirin, and linsitinib.

**Conclusions:**

Collectively, our findings suggest that PRDM14 is a positive regulator of gastric cancer progression. Therefore, it may be a potential therapeutic target for gastric cancer.

**Supplementary Information:**

The online version contains supplementary material available at 10.1186/s12885-024-12424-1.

## Introduction

Gastric cancer (GC) is a prevalent gastrointestinal malignancy, ranking fifth in incidence and fourth in mortality worldwide [1]. Despite advancements in early screening and anti-cancer strategies, late-stage diagnoses often result in poor outcomes marked by metastasis and chemotherapy resistance [2]. The complex pathogenic mechanisms of GC warrant the exploration of novel biomarkers as therapeutic targets to enhance prognosis and guide individualized treatment strategies [3].

PRDM14, a member of the PRDI-BF1 and RIZ homologous (PR) structural domain (PRDM) transcriptional regulatory family, features six Cys2His2 (C2H2)-type zinc-finger (Znf) structural domains [4]. Even though the PR domains of PRDM14 is not similar to those of five PRDM family members that exhibit histone methyltransferase (HMTs) activity [5, 6], PRDM14 governs cell development, differentiation, and stem cell growth, and primordial germ cells [7–10]. Although lacking HMT enzymatic activity [11], PRDM14 may induce chromatin structure alterations, affecting DNA-protein binding by recruiting histone-modifying enzymes to target gene promoter regions with aberrant methylation [12], similar to histone methylation transferase. This mechanism likely contributes to the impact of PRDM14 on tumor formation and growth. The involvement of PRDM14 in various tumors, promoting breast carcinoma cell growth and diminishing cancer cell chemotherapeutic sensitivity, highlights its critical role in cancer development [13]. PRDM14 also facilitates non-small cell lung cancer (NSCLC) metastasis by regulating matrix metalloproteinases for extracellular matrix degradation [14]. Despite this evidence, limited information exists regarding the effect of PRDM14 on GC.

Thus, we aimed to elucidate the influence of PRDM14 on GC. We conducted functional analyses employing differentially expressed genes (DEGs) of PRDM14 and explored the interplay between PRDM14-regulated genes, immunity, and PRDM14 expression. We further developed a prognostic model to predict survival in patients with GC. Finally, we aimed to identify potential therapeutic targets for GC by predicting corresponding sensitive drugs. We believe that our findings would provide further insights into the action mechanisms of PRDM14 and help identify novel potential targets for GC treatment.

## Materials and methods

### Pan-cancer analyses

The SangerBox web tool (http://sangerbox.com/*)* was used to monitor PRDM14 expression across various cancer types. Methylation and copy number variation (CNV) data were sourced from cBioPortal (http://www.cbioportal.org/*)* and UALCAN (http://ualcan.path.uab.edu/index.html*)*, respectively. A Pearson’s correlation test was used to investigate the correlation between PRDM14 and the aforementioned factors in the pan-cancer analysis. Gastric cancer RNA sequencing data (fragments per kilobase of transcript per million mapped reads [FPKM]) were obtained from The Cancer Genome Atlas (TCGA).

### Cell lines and culture, and siRNA transfection

Human GC cell lines SNU-601, MKN-45, BGC, and MGC-803 were obtained from ATCC (Manassas, VA, US) and cultured in Roswell Park Memorial Institute (RPMI) 1640 medium (Gibco, MA, USA) containing 10% fetal bovine serum and 1% penicillin/streptomycin. The cells were maintained in a dehumidified incubator (5% CO_2_) at 37 °C. For transfection experiments, 1 × 10^5^ SNU-601 and MGC-803 cells were inoculated into 6-well plates and transfected with 10 nM siRNA-NC or 10 nM siRNA-PRDM14. Transfection was performed using a kit (Ribobio, Guangzhou 510,663, China), and the effect was validated using western blotting using an anti-human PRDM14 antibody (1:1000, Affbiotech, AB_2839325). Double-stranded *PRDM14* siRNA was prepared by Biologicals (Ribobio, Guangzhou 510,663, China) and selected to correspond to the following DNA target sequence 5′-CTCAAACTCTGGATAAAGA- 3′.

### Validation of the expression levels of related regulatory genes in cell lines

Cell lines were pretreated to extract total RNA, and cDNA was synthesized using a Prime Script RT kit (639,505, TaKaRa, Japan) following the manufacturer’s instructions. Total RNA was extracted using qPCR SYBR Green Master Mix (Hieff). Quantitative PCR was performed using a real-time PCR system to analyze the expression levels of PRDM14 and the relevant regulatory genes. The primer sequences for the validated genes are listed in Supplementary Table S1. The levels of transcriptional statistical analysis of GAPDH were used for normalization. The relative mRNA expression levels of the target genes were calculated using the 2-ΔΔCT method [15].

### Cell proliferation and migration assay

Cancer cell line (SNU-601 and MGC-803) suspensions were seeded into 96-well plates, cell concentration was adjusted to 8,000 cells each well. 100 µL 10% Cell Counting Kit-8 (CCK-8) was mixed in each well the next day. The supernatant was aspirated into a new 96-well plate for incubation, and the OD value at 450 nm was subsequently determined using a microplate reader (Bomei Biotechnology, Nanning, China). Five replicate wells were used for each experimental group, and the results were derived from two independent experiments. Cell migration assays were performed two days after transfecting cells with si-PRDM14 or control siRNA. A straight line was drawn with a marker pen horizontally across the back of the 6-well plate, with at least five lines crossing each well. SNU-601 and MGC-803 cells were seeded in each well and scratched on the next day in a straight line perpendicular to the back of the culture plate. The cells were islodged, cultured, and observed. All experiments were conducted in triplicate for three independent trials.

### Flow cytometry analysis of cell cycle and apoptosis

For the cell cycle analysis, 400 µL propidium iodide (PI) buffer and 100 µL RNase A were added and incubated for 30 min at 4 °C. The cell cycle was measured using flow cytometry (Becton Dickinson, San Diego, CA, USA). and analyzed using ModFit software. For apoptosis, GC cells were double-stained with Annex V-FITC/PI using the eBioscience and Annexin V apoptosis detection kits (88-8005-74, Thermo Fisher Scientific, USA). The cells were resuspended at a density of 1 × 106 cells/mL in binding buffer. 100 µL of the resultant solution was added into culture tubes together with 5 µL of FITC Annex V and 5 µL of PI. Following a 15-minute dark incubation period, 400 µL of binding buffer was added to each tube so that flow cytometry could analyze the combination.

### Screening PRDM14-related DEGs and functional enrichment analysis

Patients with STAD were categorized into high and low expression groups based on the median PRDM14 expression value to identify PRDM14-related genes. DEGs between these groups were identified using the “limma package” [16]. Significance criteria for PRDM14-associated genes were set at |fold-change| > 1.5 and an adjusted p-value < 0.05. Gene Ontology (GO) and Kyoto Encyclopedia of Genes and Genomes (KEGG) pathway enrichment analyses for differentially expressed PRDM14-related genes were conducted using the “ClusterProfiler” package [17].

### PRDM14-related prognostic and nomogram model

In the TCGA cohort, DEGs influenced by PRDM14 were estimated using univariate Cox regression analysis. Genes with a p-value < 0.05 were identified as prognostic factors and entered into LASSO regression analysis. The selected genes were included in a multivariate Cox regression model, leading to the calculation of the PRDM14-associated risk score model using the formula: $riskscore=\sum _{k-1}^{n} Ex{p}_{i}^{ }\ast {coef}^{HR}\text{i}$. Patients with GC were stratified into high- and low-risk subgroups based on median risk scores. The “ggrisk” package [18] was used to visualize the risk score distribution, survival status, and expression heatmap of signature genes. Kaplan-Meier curves for OS between high and low-risk subgroups were plotted using the “survival” and “survminer” packages. Receiver operating characteristic (ROC) curves for 1-, 3-, and 5-year survival were created using the survival ROC package, and the area under the curve (AUC) was calculated [19]. The prognostic value of the PRDM14 differential genomic model was externally validated in the GSE62254 cohort.

### Univariate and multivariate Cox regression models

To assess the relationship between clinical indicators (age, sex, stage, T, N, and M), PRDM14-related risk scores, and OS in patients with GC, univariate and multivariate Cox regression models were developed from TCGA and GSE62254 data. The predictive power of the screened clinical prognostic model was assessed using ROC curves. A multifactorial analysis, based on a regression analysis nomogram prediction model, was employed. Calibration curves were used to evaluate the agreement between actual and nomogram-predicted survival probability.

### Prediction of drug response

The Genomics of Drug Sensitivity in Cancer (GDSC) project (https://www.cancerrxgene.org/*)* provides drug sensitivity information for 138 anti-cancer drugs in approximately 75,000 experiments using 700 cancer cell lines [20]. The half-maximal inhibitory concentrations (IC50) representing the drug response were estimated using the pRRophetic package [21]. Upregulated and downregulated DEGs were uploaded to the cMAP database (https://clue.io*)* to identify potential drugs for the treatment of GC [22] and matched with small-molecule therapies. Four important small-molecule drugs and their enrichment scores were listed. Correlation scores (− 100 to 100) were obtained based on the enrichment of DEGs in the reference gene expression profile.

### Statistical analysis

Statistical analyses were performed using R software (v4.2.1; https://www.r-project.org/). The assumptions of the t-test, including normality of data, were assessed, and met. Comparisons between groups were conducted using the Student’s t-test or the Wilcoxon rank-sum test. Pearson’s correlation test was employed to determine interactions between variables. Statistical significance was set at *P* < 0.05.

## Results

### Overexpression of PRDM14 in gastric cancer

**Fig. 1:**
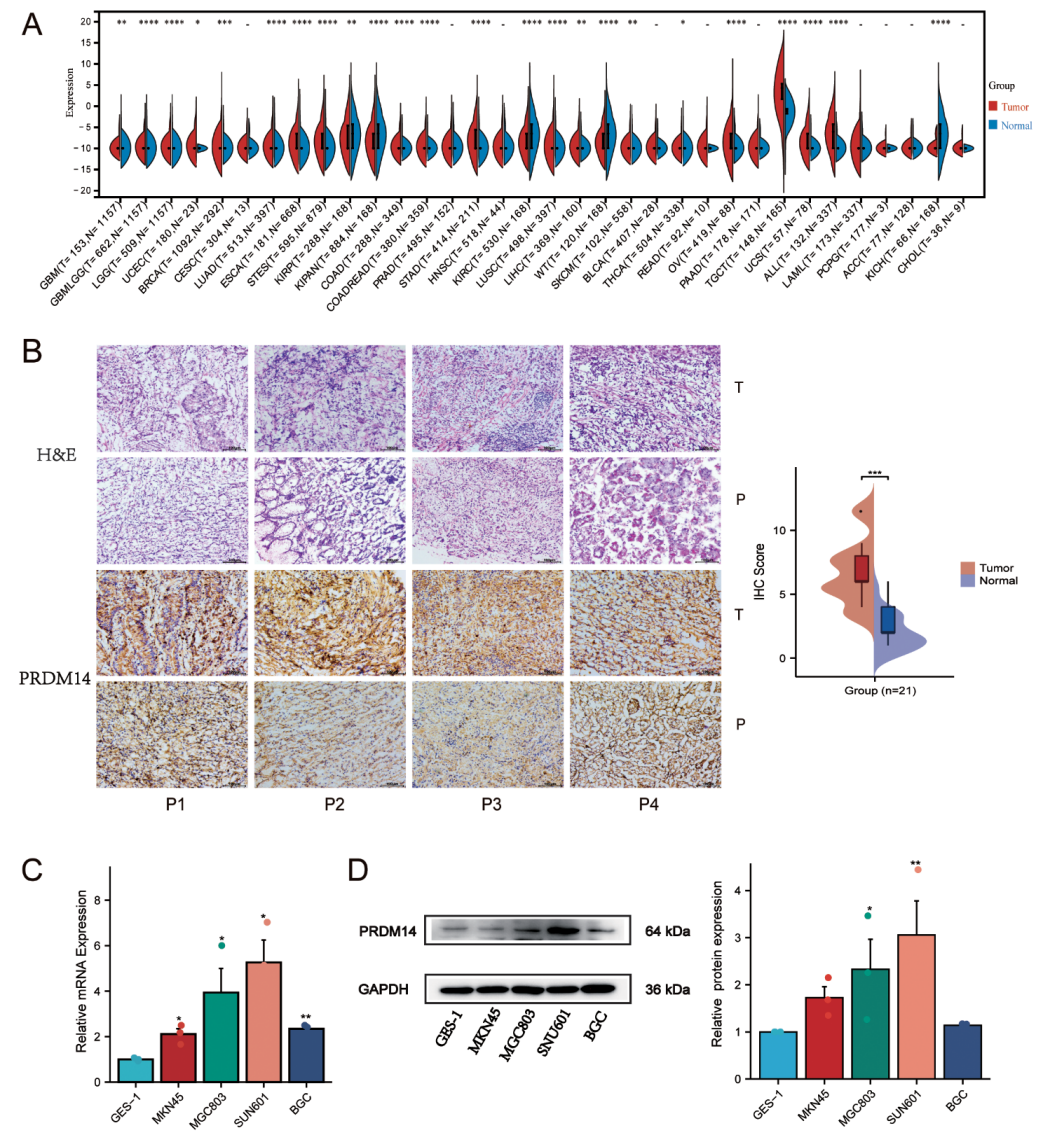
PRDM14 overexpression in gastric cancer cells and patients. (**A**) The expression of PRDM14 in TCGA pan-cancer. (**B**) Comparison of PRDM14 expression in carcinoma mucosa and paired primary GCs with immunohistochemistry, T: Tumor; P: Paratumorous tissue, Scale bar: 200 mm. The mRNA (**C**) and protein (**D**) expression of PRDM14 in four GC cell lines and gastric epithelial histiocytic cell line. GC cell lines: MKN45, MGC803, SNU601, BGC; gastric epithelial histiocytic cell line: GSE-1

In general, PRDM14 expression was not noteworthy. However, its expression was significantly increased in certain cancers (*p* < 0.05, Fig. 1A), including low-grade glioma (LGG), breast invasive carcinoma (BRCA), lung adenocarcinoma (LUAD), esophageal carcinoma (ESCA), stomach adenocarcinoma (STAD), and colon adenocarcinoma (COAD). We observed that patients with high PRDM14 expression were accompanied by pan-cancerous genetic alterations (Supplementary Figure S1A). There is a positive correlation between PRDM14 expression with CNA (*p* < 0.01, Supplementary Figure S1B) and DNA promoter methylation levels in STAD (*p* < 0.01, Supplementary Figure S1D). However, we observed no correlation between PRDM14 expression and mutations, as well as global methylation in STAD (*p* > 0.05, Supplementary Figure S1C). Collectively, our findings suggest that PRDM14 is differentially expressed in most tumors, genotypic changes may be the source of differential PRDM14 expression, and that PRDM14 is abnormally active in GC.

We monitored PRDM14 expression in tumor tissues from patients using immunohistochemical analysis, and observed that its expression was significantly higher in GC tissues than in paraneoplastic tissues (*p* < 0.001) (Fig. 1B). We further examined the expression levels of PRDM14 in four GC cell lines (MKN-45, MGC-803, SNU-601, and BGC) and gastric epithelial cells (GES-1). The results suggested that the mRNA expression level of PRDM14 was higher in BGC, MKN-45, MGC-803, and SNU-601 (*p* < 0.05, Fig. 1C) and protein level was higher in MGC-803 and SNU-601 (*p* < 0.05, Fig. 1D). Regretfully, we found no discernible variation in PRDM14 expression regardless of stage(A), molecular subtype(B), Lauren categorization(C), or lesion location(D) of GC (Supplementary Figure S2). Overall, PRDM14 is more active in various tumor genomics, but its regulatory mechanism and impact on tumors are unclear, such as gastric cancer.

### Silencing PRDM14 inhibits proliferation, migration, cell cycle, and promotes apoptosis in GC cells

**Fig. 2:**
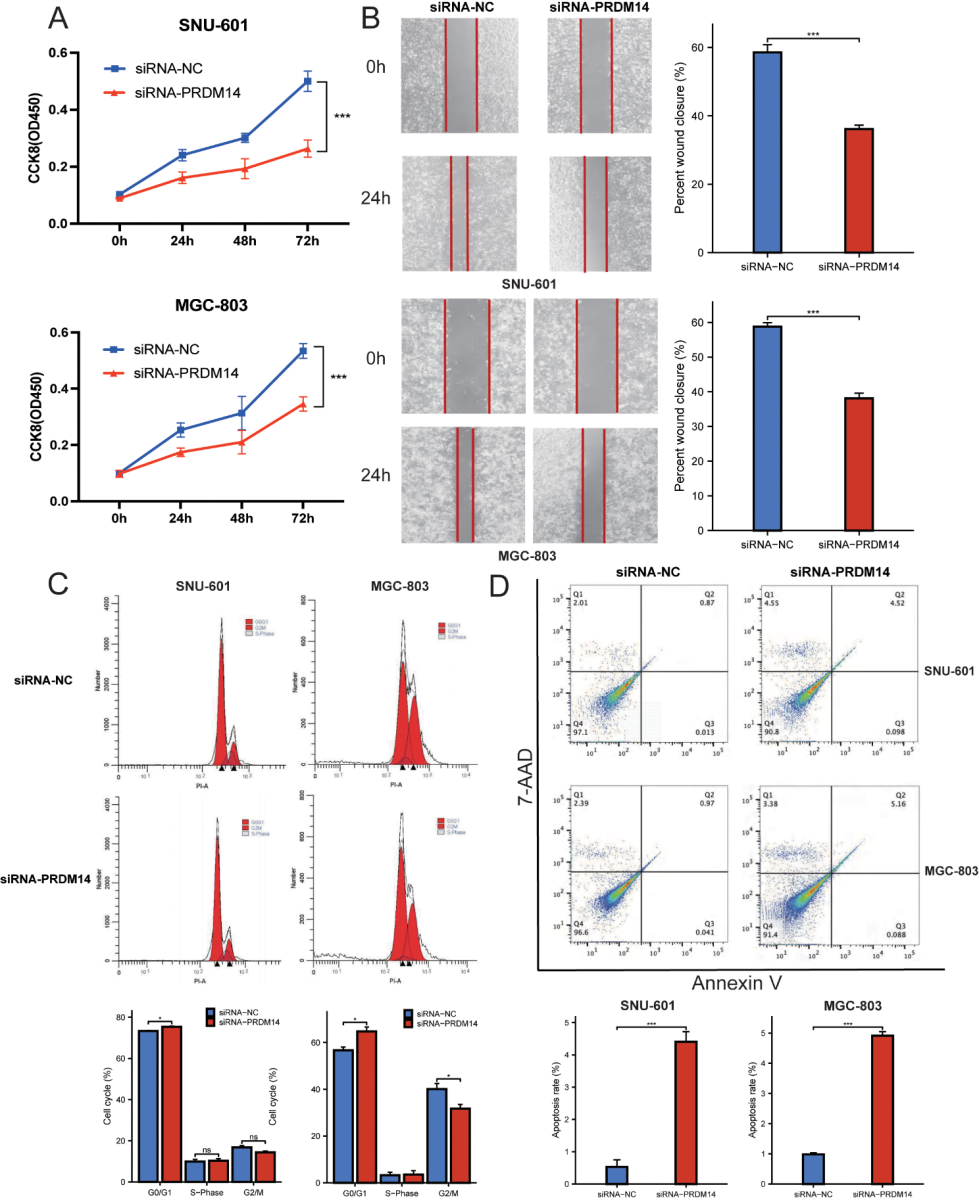
Silencing PRDM14 inhibits proliferation, migration, cell cycle, and promotes apoptosis in GC cells. (**A**) Cell proliferation analysis with siRNA-PRDM14 cell activity at different times (0 h, 24 h, 48 h, and 72 h). (**B**) Cell migration of SNU-601 and MGC-803 cells after siRNA treatment. (**C**) Cell cycle distribution of SNU-601 and MGC-803 cells after siRNA application. (**D**) Apoptosis of SNU-601 and MGC-803 cells at various stages after siRNA treatment

To investigate the effect of PRDM14 on gastric cancer cells, we used small-interfering RNA (siRNA) to silence PRDM14 expression in SNU-601 and MGC-803 cells (Supplementary Fig. 5). The cellular activity of both GC cell lines (SNU601 and MGC803) was significantly reduced when transfected with PRDM14 siRNA (Fig. 2A, Supplementary Fig. 6A, *p* < 0.001). And the GC cells’ migration were significantly reduced in both SNU601 and MGC803 cell lines when transfected with PRDM14 siRNA (Fig. 2B, Supplementary Fig. 6B, *p* < 0.001). The cell cycle assays also demonstrated that silencing PRDM14 expression influenced GC cell cycle arrest. The percentage of G0-G1 phase cells was significantly higher in SNU-601 and MGC-803 cells in the siRNA-PRDM14 group compared to the siRNAs-NC group (Fig. 2C, Supplementary Fig. 6C, *p* < 0.05), although the percentage of S phase cells did not significantly change. Compared with the siRNA NC group, the apoptosis rate of GC cells showed a significant increase (Fig. 2D, Supplementary Fig. 6D, *p* < 0.001). In summary, inhibiting the expression of PRDM14 can significantly inhibit the characteristics of tumor cells.

### Identification of PRDM14-related functional enrichment

**Fig. 3:**
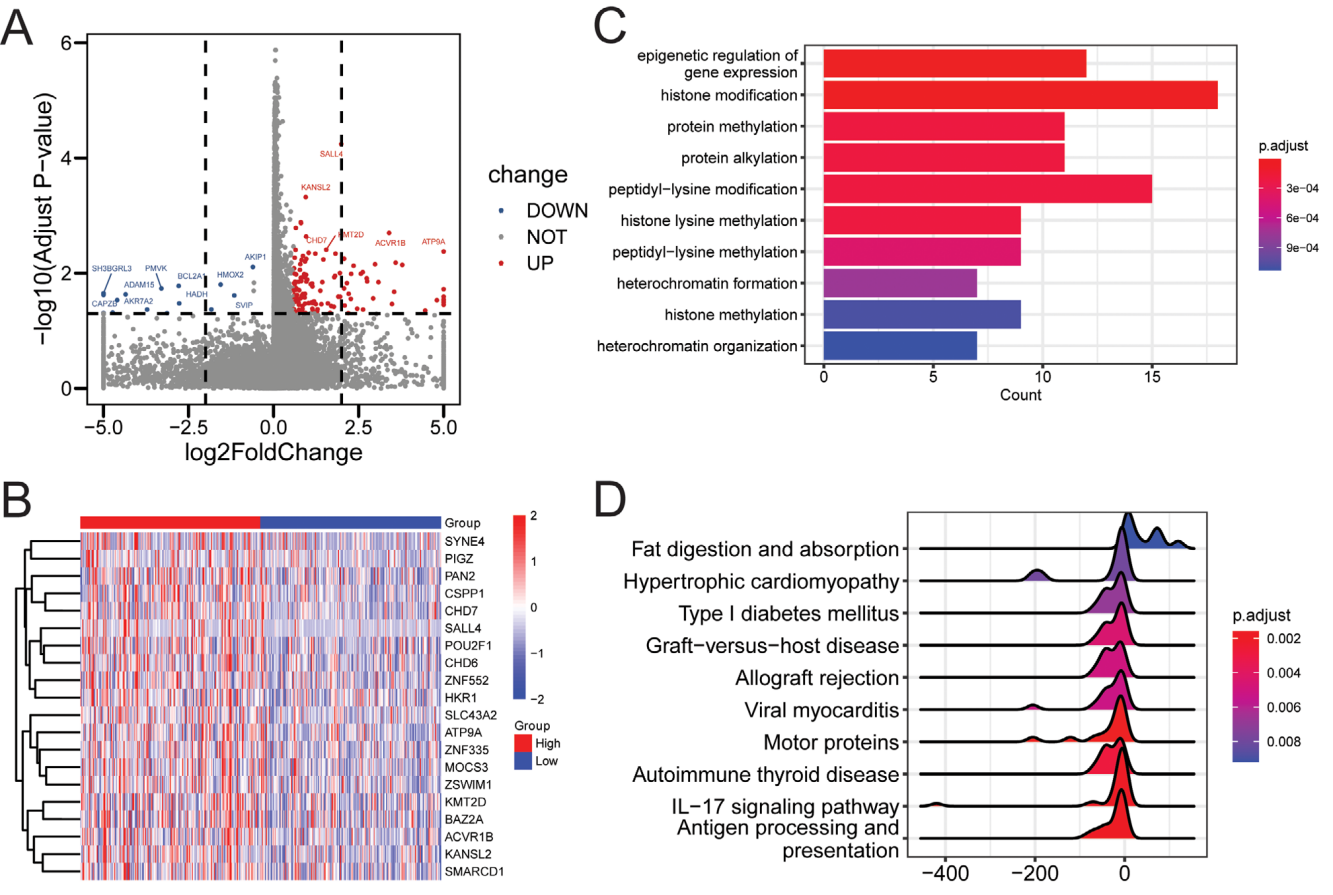
Demonstration of PRDM14-related differentially expressed genes and function analysis. (**A**) Volcano plot of 146 PRDM14-related differential genes. (**B**) Heatmap of expression of the top 20 PRDM14-related differential genes. (**C**) Gene Ontology-Biological Processes enrichment analyses of DEGs. (**D**) Gene set enrichment analysis for the signaling pathways activated by DEGs

PRDM14 can significantly affect the characterization of gastric cancer cells, therefore it is worth further exploring the function of PRDM14 in gastric cancer. We identified 146 PRDM14 DEGs using a cutoff value of |fold-change| > 1.5 and an adjusted p-value of < 0.05, 131 of the 146 DEGs were upregulated, and 15 were downregulated (Fig. 3A, Supplementary Table S2). The expression of the top 20 DEGs is presented in the heatmap (Fig. 3B). We further analyzed the functions involving PRDM14. We observed that these DEGs are usually closely associated with the epigenetic regulation of gene expression, histone modifications, and associated methylation functions (Fig. 3C). Gene set enrichment analysis (GSEA) results revealed that DEGs for PRDM14 were commonly enriched in antigen processing and presentation, IL-17 signaling pathways, motor proteins, nutritional metabolic processes, and immune systemic disease pathways (Fig. 3D). Therefore, we found that PRDM14 plays a variety of functions in cancer, and consistent with previous studies, methylation modification is directly influenced by PRDM14, while other functions may be indirectly affected.

### PRDM14 regulates the expression of methylation target genes

**Fig. 4:**
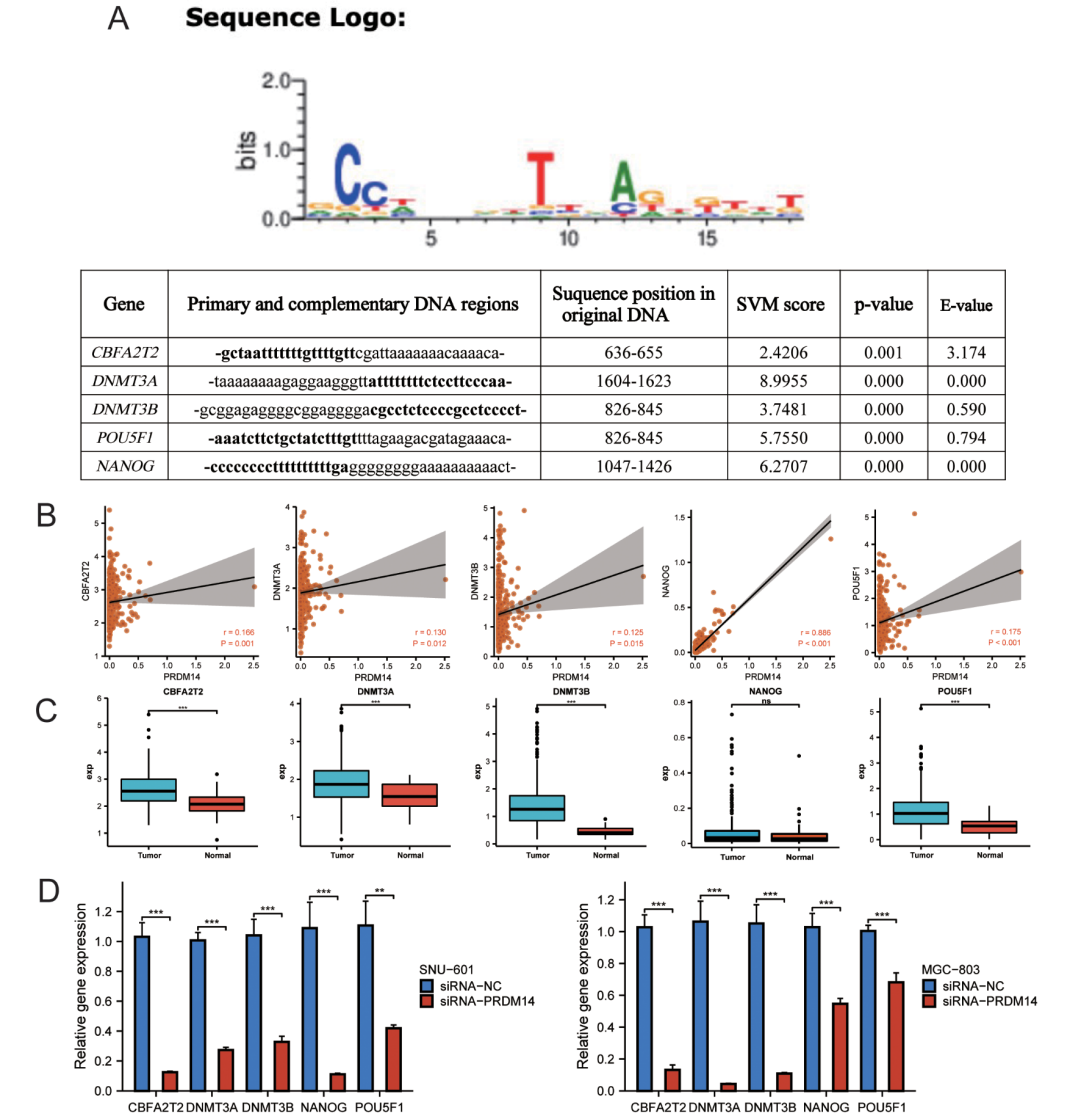
PRDM14 regulates the expression of methylation target genes. (**A**) Predicted DNA binding sites of PRDM14 and associated regulatory genes, SVM score: support vector machine. (**B**) Correlation analysis of PRDM14 and associated regulatory genes in TCGA. (**C**) Differential analysis of associated regulatory genes between tumor and normal tissues in TCGA. (**D**) si-PRDM14-mediated expression of associated regulatory genes

Then we investigated the effect of PRDM14 on methylation target genes in gastric cancer. Then we reviewed relevant studies and identified some target genes closely related to PRDM14, including CBFA2T2, DNMT3A, DNMT3B, POU5F1, and NANOG. We used the DNA binding site prediction website (http://zf.princeton.edu/) to verify the binding region between the PRDM14 zinc-finger structure and target genes (*p*-value < 0.001, Fig. 4A). And the PRDM14 expression was closely and positively correlated with the expression of related genes (*p* < 0.05; Fig. 4B). Meanwhile, the expression levels of *DNMT3A*, *DNMT3B*, *CBFA2T2*, and *POU5F1* were significantly upregulated in tumor tissues (*p* < 0.001, Fig. 4C), whereas that of NANOG showed no difference. Finally, qPCR analysis to validate the related regulatory genes post-PRDM14 silencing in SNU-601 and MGC-803 cells revealed that the levels of *DNMT3A*, *DNMT3B*, *CBFA2T2*, and *POU5F1* were significantly reduced in the si-PRDM14 cells (*p* < 0.01, Fig. 4D, Supplementary Fig. 7).

### Construction of a PRDM14-related prognostic and nomogram model

**Fig. 5:**
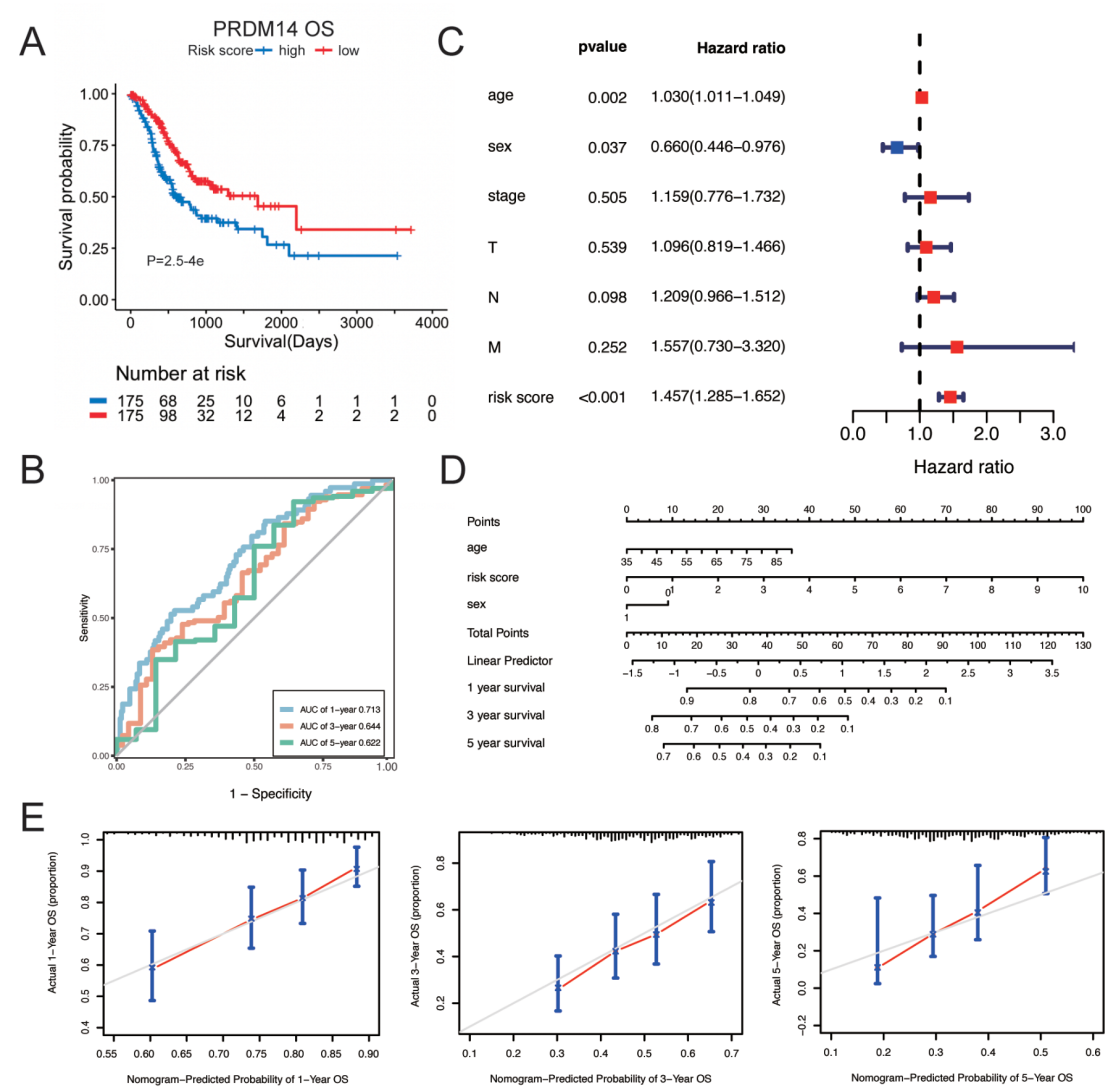
Construction of a PRDM14-related prognostic and nomogram model for gastric cancer. (**A**) Kaplan-Meier curves of overall survival for high and low risk score subpopulations. (**B**) Receiver operating characteristic curves at 1-, 3-, and 5-year OS outcomes in accordance with PRDM14-differential risk score. (**C**) Multivariate Cox regression models were developed to elucidate the association of clinical features and risk score with GC survival outcome. (**D**) A nomogram was developed by integrating independent prognostic indicators: age, sex, and PRDM14-differential risk score. (**E**) Calibration plots displaying the association of predicted 1-, 3-, and 5-year OS with actual survival duration

We constructed a prognostic model of PRDM14 related genes to further explore the impact of PRDM14 on patients’ prognosis. LASSO regression analysis was used to define the final selected genes from the PRDM14-related DEGs (Supplementary Fig. 3). In addition, a multivariate Cox regression model was developed with the formula: risk score = -0.0782692×exp_PATJ_ -0.0530962 ×exp_STK36_ + 0.0526597×exp _GRB10_ + 0.0791429×exp_ZBTB10_ − 0.0391334×exp_AKIP1_ -0. 0147172×exp_SLC11A2_ +0.0956068×exp_KLHDC2_ + 0.0050631 ×exp_TTR_ + 0.0020701×exp_GNAS_ -0.1279056×exp_HAUS5_ -0.0638204×exp_KMT5C_. The Kaplan-Meier plot revealed that low-risk GC individuals demonstrated a high survival advantage (*p* < 0.001, Fig. 5A). The receiver operating characteristic (ROC) curves confirmed that the PRDM14-differentiated genomic model had overall survival probabilities of 0.713, 0.644, and 0.622 at 1, 3, and 5 years, respectively (Fig. 5B). We classified GC patients into high- and low-risk subgroups using the average values (Supplementary Fig. 4A) and observed a significant difference in survival between the high- and low-risk subgroups (Supplementary Fig. 4B). Based on this, the expression of most genes, including STK36, GRB10, ZBTB10, KLHDC2, and KMT5C, was upregulated in the high-expression group (Supplementary Fig. 4C).

Univariate and multivariate Cox regression analyses revealed that age, sex (0: male 1: female), and risk score (age and risk score: HR > 1, sex (HR < 1), *p* < 0.001) were independent prognostic indicators for GC (Supplementary Fig. 4D and Fig. 5C). By integrating these prognostic indicators, the risk score contributed the most to predicting OS duration at 1, 3, and 5 years. We utilized a nomogram to estimate the survival outcomes of GC patients (Fig. 5D). In addition, we evaluated the predictive performance of the nomogram using calibration curves. Our data revealed that the 1-, 3-, and 5-year survival rates predicted by this nomogram were close to the actual survival rates (Fig. 5E).

**Fig. 6:**
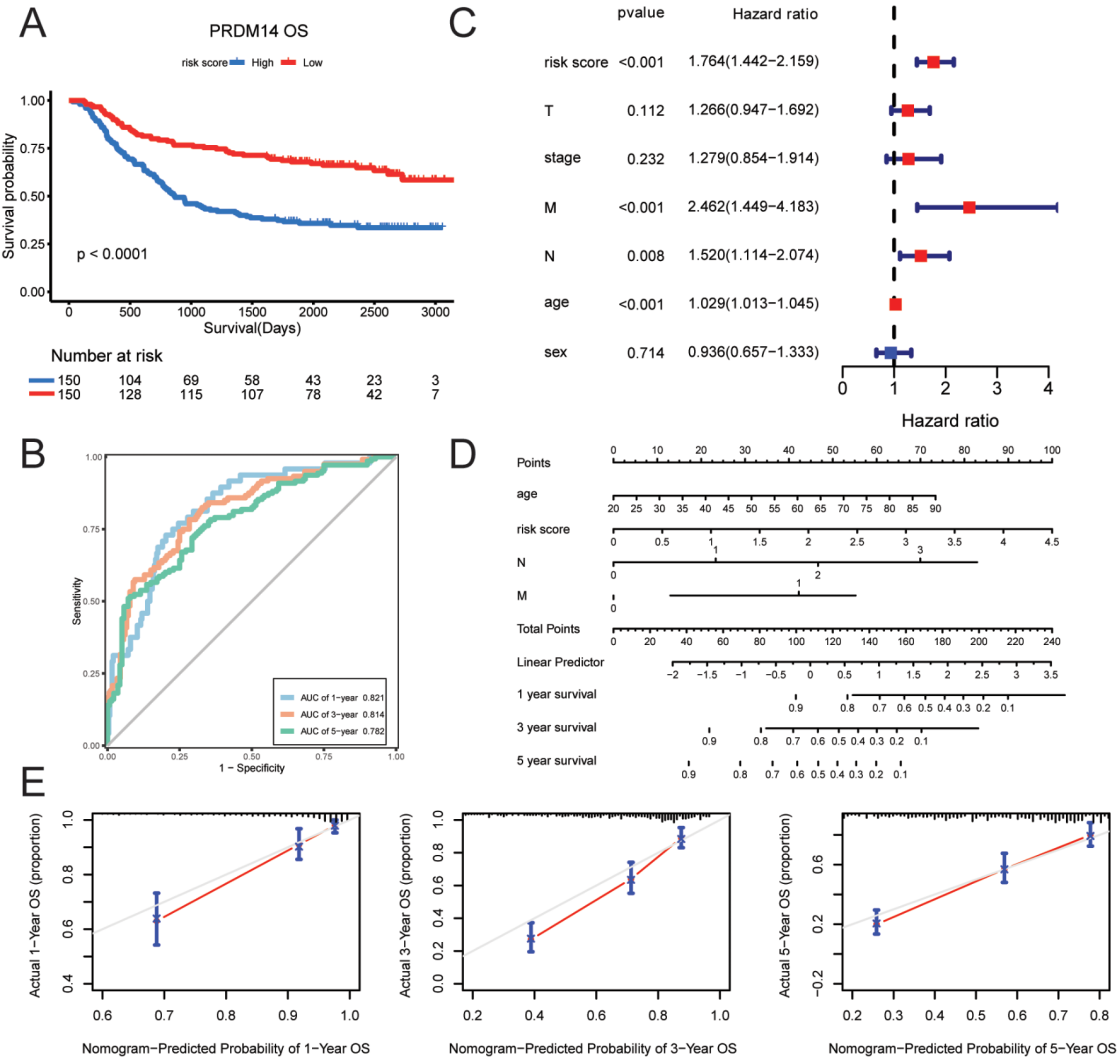
Validation of prognostic significance in gastric cancer validation cohort GSE62254. (**A**) Kaplan-Meier curves of overall survival (OS) for high and low GC subpopulations in the GSE62254 dataset. (**B**) Multivariate Cox regression models revealed the correlation between clinical characteristics and risk score. (**C**) Time-dependent receiver operating characteristic analysis of risk score depicting the overall survival (OS) of patients. (**D**) A nomogram was developed by integrating independent prognostic indicators (age, N, M, and risk score). (**E**) Calibration plots displaying the association of predicted 1-, 3-, and 5-year OS with actual survival duration

We also validated this prognostic model in an external cohort. The high-risk group was predicted to have favorable OS outcomes in the validation dataset (*p* < 0.001, Fig. 6A). The area under the curve (AUC) values of the risk score model (OS at 1, 3, and 5 years: 0.821, 0.814, and 0.782, respectively) exhibited excellent efficiency (Fig. 6B). The multivariate Cox regression analysis was performed to screen for age, M, N, and risk score (HR > 1, *p* < 0.001) as independent prognostic indicators for GC (Fig. 6C). Subsequently, a nomogram was constructed using these four metrics, and the survival outcomes of GC patients were assessed using the nomograms (Fig. 6D). We confirmed that the 1-year and 5-year survival rates predicted using the nomogram were close to the actual survival rates, whereas the prediction of the 3-year survival was unsatisfactory (Fig. 6E). These results suggest the predictive power of this nomogram for the risk score.

### Screening small-molecule drugs and assessing drug sensitivity

**Fig. 7:**
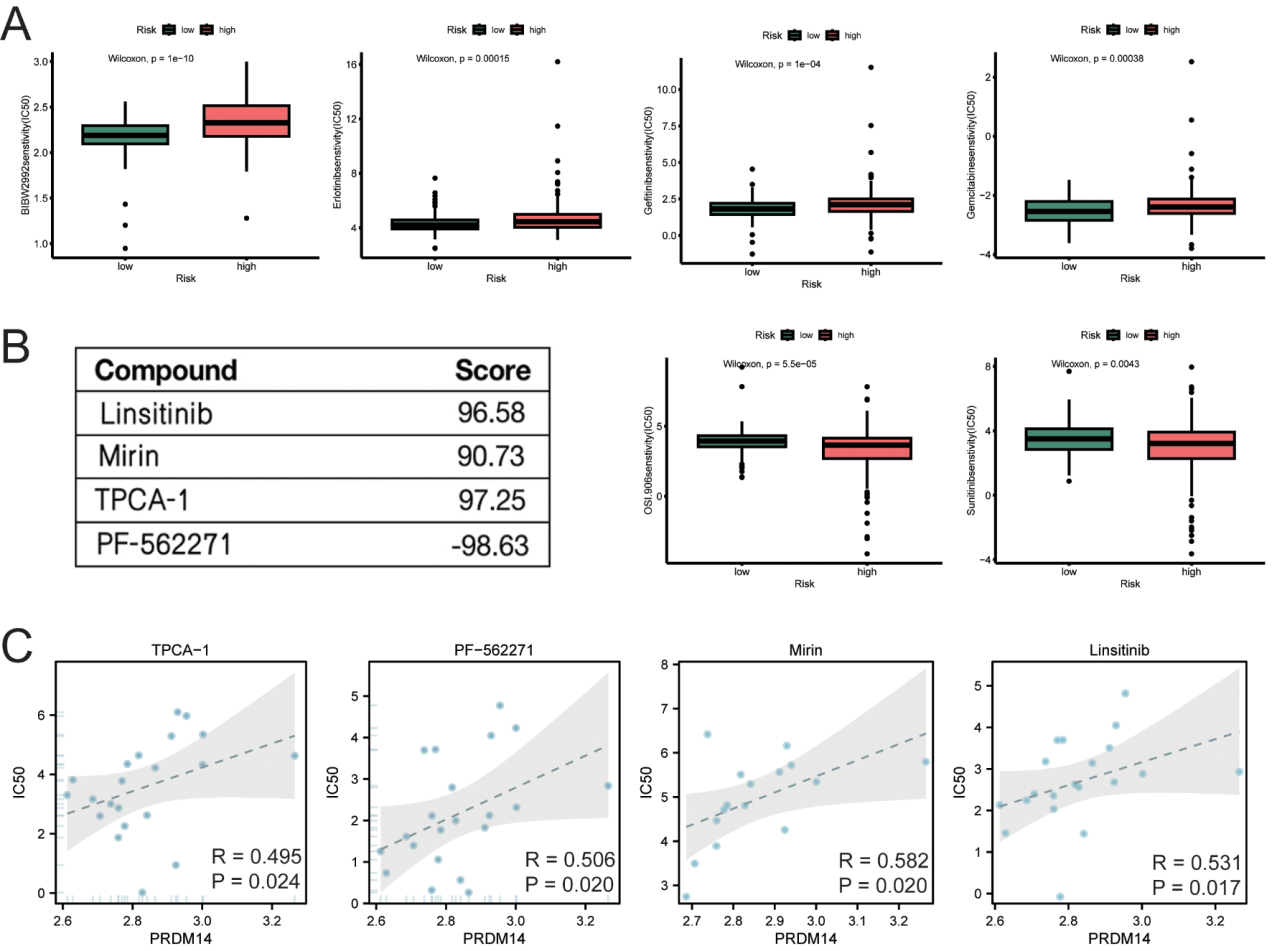
Association of PRDM14 with drug sensitivity in gastric cancer. (**A**) Comparison of the estimated IC_50_ values of afatinib, erlotinib, gemcitabine, bexarotene, gefitinib, and linsitinib between high and low-risk score GC subgroups. (**B**) Four small-molecule drugs with their corresponding enrichment fractions in cMAP. (**C**) Scatter plots displaying the correlation of PRDM14 expression with the drug sensitivity of four small-molecule drugs in gastric cancer

To discover the potential of PRDM14 as a drug target, we explored potential therapeutic drugs for PRDM14 through drug sensitivity analysis. The estimated IC_50_ values for bexarotene and linsitinib were significantly lower in the high-risk group than that in the low-risk group, with lower estimated IC_50_ values for erlotinib, gemcitabine, afatinib, and gefitinib in the low-risk group, indicating that the low-risk subgroup was more likely to respond to erlotinib, gemcitabine, afatinib, and gefitinib (Fig. 7A). The correlation between PRDM14 expression and drug sensitivity was explored using the Connectivity Map(cMAP) database. Four small-molecule drugs with absolute enrichment scores greater than 90 were selected for demonstration, and PRDM14 positively correlated with sensitivity to TPCA-1, PF-56,227, mirin, and linsitinib (Fig. 7B and C).

## Discussion

Gastric cancer is a widespread and highly prevalent tumor worldwide that is biologically and genetically heterogeneous [23]. The pathological mechanisms of its initiation and progression are associated with various molecular aberrations [24, 25]. The PRDM family is a transcriptional regulatory family involved in human tumorigenesis that plays a vital role in cell differentiation and malignancy [26]. PRDM14 is mainly expressed in primordial germ cells and certain pluripotent stem cells to reset and maintain cellular pluripotency [27, 28]. We identified the expression of PRDM14 mRNA levels in various tumors in TCGA data, but high or low expression was not consistent. Many studies shown that PRDM14 is overexpressed in pancreatic, breast, and non-small cell lung cancers [13, 29, 30], but it is not well understood in gastric cancers. Our results shown that the mRNA and protein expression levels of PRDM14 are overexpressed in gastric cancer tumor cell lines and patient tissues, which indicate that PRDM14 plays a role as an oncogene in gastric cancer.

PRDM14 has been confirmed to be associated with the degree of differentiation and tissue type [14, 31, 32]. The PRDM14 expression level significantly increased with the hyperdifferentiation of cancer cells, and occur in the early stages of cancer while promoting cell proliferation [8]. We constructed siRNA-PRDM14 and performed cell phenotyping experiments to corroborate the effects of PRDM14 on tumor cells. Silencing the expression of PRDM14 resulted in apoptosis promotion, cell cycle arrest, and inhibition of the growth and migration of GC cells. Functional analysis of DEGs related to PRDM14 showed that PRDM14 was associated with epigenetic regulation, histone modifications, and methylation. Similarly, promoter hypermethylation may contribute to cancer development by silencing tumor suppressor genes that are involved in the control of tumor-specific signaling pathways, DNA repair, the cell cycle, and apoptosis. Therefore, elevated expression of PRDM14 may contribute to cancer development by promoting epigenetic reprogramming, self-renewal, and pluripotency of somatic cells [33]. PRDM14 is inextricably associated with GC tumorigenesis.

DNA hypermethylation leads to genomic instability [34]. Moreover, PRDM14 plays a role in genome-wide DNA demethylation. PRDM14 binds to PRC2 and forms a repressive H3K27me3 protein complex that inhibits the DNA methyltransferases Dnmt3a, Dnmt3b, and Dnmt3l in mouse embryonic stem cells (mESCs) [9, 35]. DNMT gene family members DNMT3A and DNMT3B are widely upregulated in GC [36] and significantly reduced in the si-PRDM14 cells, which is consistent with our findings. However, the positive correlation between PRDM14 and DNMT family genes and the regulatory mechanism of PRDM14 in tumors requires further experimental evidence. And the small number of immune factors associated with PRDM14 expression also suggests that PRDM14 plays a small role in the immune microenvironment. However, whether PRDM14 can serve as a target for disease immunotherapy remains to be determined.

Approximately 60% of PRDM14 binding sites are located 10 kb from the transcription start site (TSS) [37], and the binding region binds to various TF promoters [38]: 42.8% to NANOG, 13.7% to POU5F1, and co-localizes with SOX2 [35]. In human embryonic stem cells(hESCs), PRDM14 activates POU5F1 through proximal enhancers, recruits it to the promoters of multiple genes, activates pluripotency networks through promoter demethylation, and recruits other TFs [39]. POU5F1 (also known as OCT4), which is an encoding factor for pluripotent transcription, affects proliferation, invasion, and metastasis in gastrointestinal cancers through different signaling pathways [40]. CBFA2T2 is a novel co-blocker protein, and CBFA2T2–PRDM14 protein interactions stabilize chromatin and repress genes involved in differentiation [41]. In the study of the target genes mentioned above, our results suggest that PRDM14 expression positively correlated with that of CBFA2T2, NANOG, and POU5F1. Although CBFA2T2 and POU5F1 levels were significantly increased in tumors, no significant differences were observed for NANOG. However, many studies show that NANOG levels are significantly elevated in primary GC compared to adjacent normal tissues [42–45]. Notably, NANOG expression was significantly downregulated after the knockdown of PRDM14 expression, suggesting a regulatory relationship between PRDM14 and NANOG expression. Thus, the interaction between PRDM14 and its target genes regulates the balance of pluripotency maintenance and renewal mechanisms in pluripotent stem cells.

We constructed a prognostic model to identify 146 PRDM14-derived genes in GC. We developed a PRDM14-derived genomic model using univariate and multivariate Cox regression analyses that reliably and independently predicted patient progression and outcomes. Nomograms used to integrate different risk factors to quantify in clinical settings [46]. The actual survival rate and the expected survival rate agreed well, and external data further validated the model’s predictive usefulness. At last, drug validation found that PRDM14 was positively correlated with sensitivity to certain small-molecule drugs. Perhaps PRDM14 will provide novel approaches to treating GC.

## Conclusion

Overall, we observed that PRDM14 is an important cancer-promoting factor for GC and a predictor of therapeutic response to GC treatment. PRDM14 is overexpressed in GC cells and patients, and interference with PRDM14 can inhibit the progression of GC, providing new ideas for the treatment of GC. Future research should provide more experimental evidence to reveal the function of the determined PRDM14-related genes in GC.

### Electronic supplementary material

Below is the link to the electronic supplementary material.

Supplementary Material 1

Supplementary Material 2

## Data Availability

The datasets analyzed for this study can be found on the TCGA-STAD project (http://www.cancer.gov/tcga) and GEO (https://www.ncbi.nlm.nih.gov/geo/query). Original data referenced in the study are included in the article/supplementary materials, and further inquiries can be directed to the corresponding author.

## Abbreviations

AUC: Area under the curve
BRCA: Breast invasive carcinoma
CCK-8: Cell Counting Kit-8
CNV: Copy number variation
COAD: Colon adenocarcinoma
DEG: Differentially expressed gene
ESCA: Esophageal carcinoma
FPKM: Fragments per kilobase of transcript per million mapped reads
GC: Gastric cancer
GDSC: The Genomics of Drug Sensitivity in Cancer
GO: Gene Ontology GSEA, gene set enrichment analysis
HMT: Histone methyltransferase
IHC: Immunohistochemistry
K-M: Kaplan–Meier
KEGG: Kyoto Encyclopedia of Genes and Genomes
LASSO: Least absolute shrinkage and selection operator
LGG: Low-grade glioma
LUAD: Lung adenocarcinoma
NSCLC: Non-small cell lung cancer
OS: Overall survival
PI: Propidium iodide
ROC: Receiver operating characteristic
RPMI: Roswell Park Memorial Institute
siRNA: Small-interfering RNA
STAD: Stomach adenocarcinoma
TCGA: The Cancer Genome Atlas
TF: Transcription factor
TSS: Transcription start site
