# Supplementary material for: Integrated analysis of the role of PR/SET domain 14 in gastric cancer
Source: BMC Cancer. 2024 Jun 5;24:685. doi: 10.1186/s12885-024-12424-1 (PMC11151633; doi:10.1186/s12885-024-12424-1)
Supplement: Supplementary file 1 — Supplementary Material 1 [file 12885_2024_12424_MOESM1_ESM.docx]

**Supplementary Material**

**Methods**

**Clinical specimens and immunohistochemistry**

Formalin-fixed paraffin-embedded specimens, including primary cancer specimens, corresponding normal tissue adjacent to the cancer for immunohistochemistry (IHC), were obtained from 21 GC patients who underwent surgery between 2022 and 2023. GC patient data and tissue samples were obtained from the Henan Cancer Hospital. None of the patients in the current study were treated with radiotherapy. Human gastric cancer biopsies (0.5-1 cm3) embedded in paraffin wax were dewaxed and placed in a repair vat containing citrate buffer (pH 6.0). After boiling and cooling, samples were blocked with 3% H2O2 for 5 minutes for endogenous peroxidase. Antibodies were incubated and then developed with the DAB substrate kit, blocked and scanned. The primary antibody used in the study included anti-human PRDM14 antibody (1:1000, affbiotech, AB_2839325). The positivity rate was calculated manually based on the extent of immunohistochemical staining. Random sections in each section were selected for analysis at a magnification of 200. All samples were reviewed by two independent and experienced pathologists who had no knowledge of the identity of the samples.

**Related regulatory genes of PRDM14**

We used an online prediction tool (http://zf.princeton.edu/)(1)to calculate the DNA bases potentially recognized by each ZNF array present at the end of PRDM14-C. For a given PRDM14 C2 H2 zinc finger protein, this website predicts a position weight matrix representing its DNA binding specificity and displays it as a sequence logo.

1. Persikov AV, Osada R, Singh M. Predicting DNA recognition by Cys2His2 zinc finger proteins. Bioinformatics. 2009;25(1):22-9. doi:10.1093/bioinformatics/btn580

**Supplementary Figures**


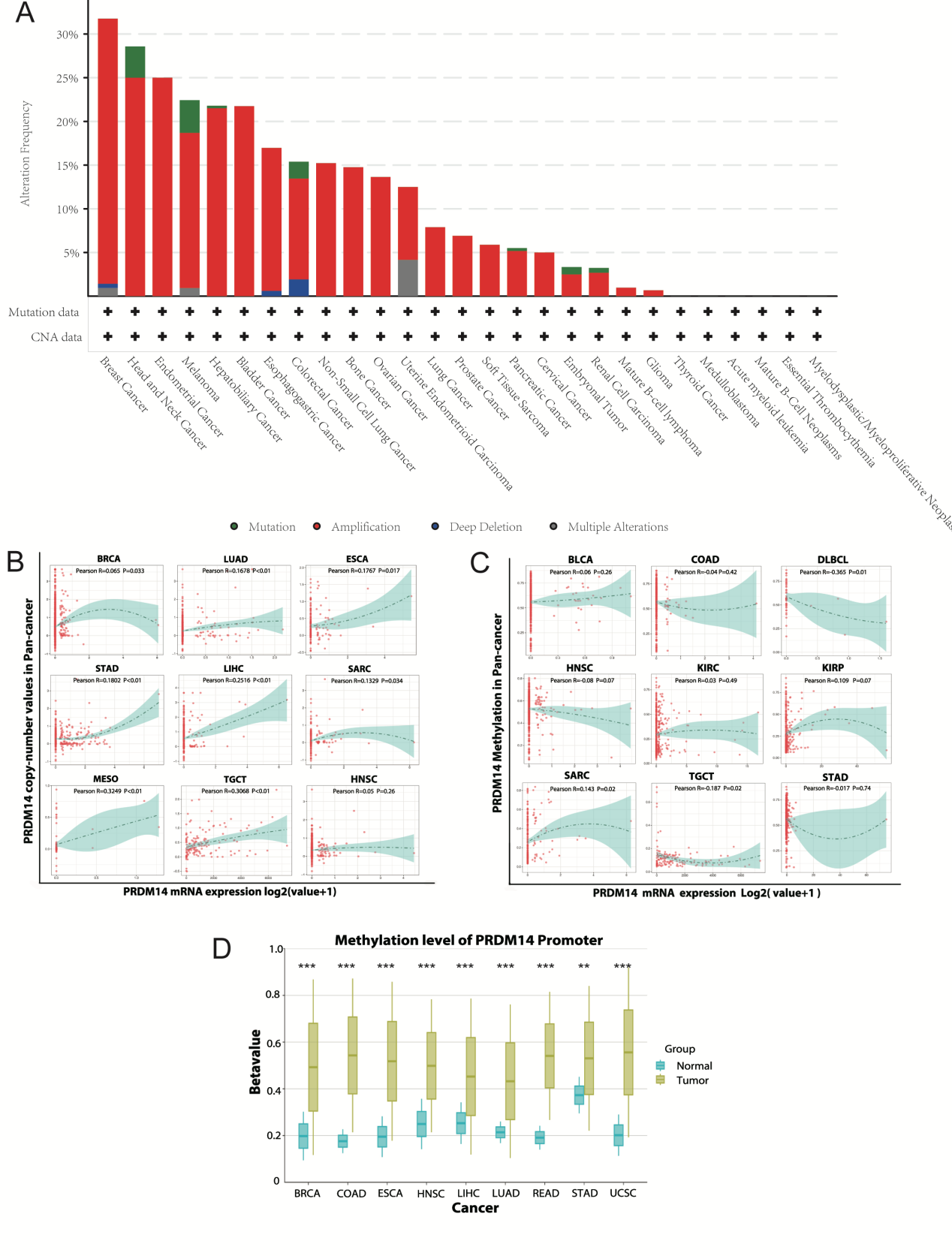


Figure S1. Analysis of CNA, and DNA methylation alterations of PRDM14 Across Pan-Cancer. (A) CNA and mutation frequency data of PRDM14 in different cancer studies were accessed from cBioPortal. (B) Positive correlations between PRDM14 expression and relative liner copy-number values. (C) The relationship between DNA methylation and mRNA expression of PRDM14. (D)DNA-methylation beta values ranging from 0 (unmethylated) to 1 (fully methylated).


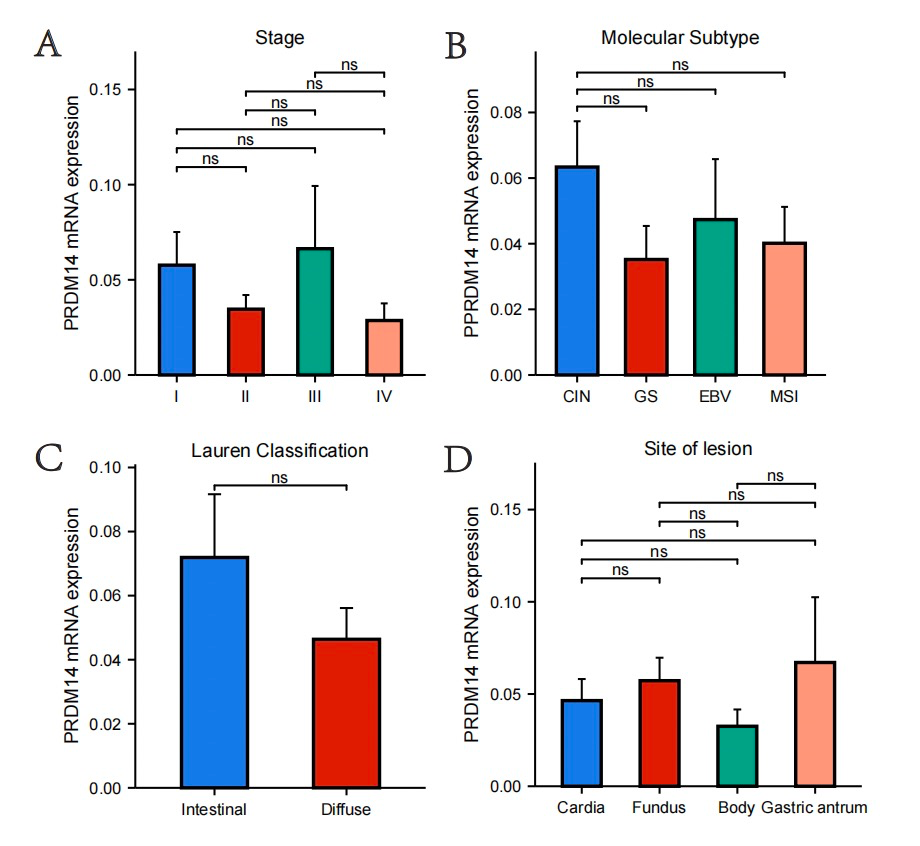


Figure S2. Relationship between PRDM14 and stage (A), molecular subtype (B), Lauren classification (C), and site of lesion (D).


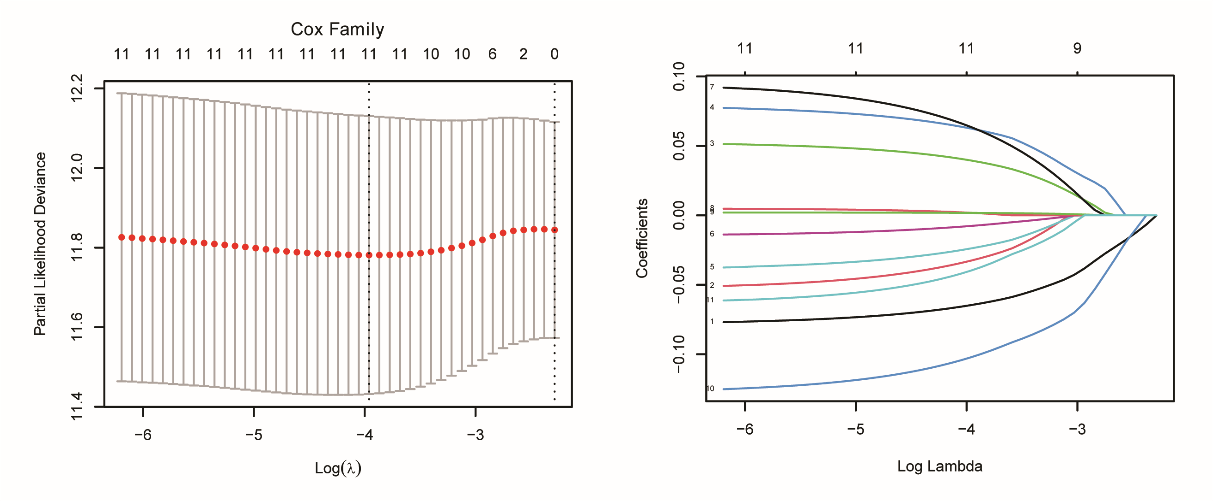


Figure S3. The lasso model screens genes to construct a multifactorial model.


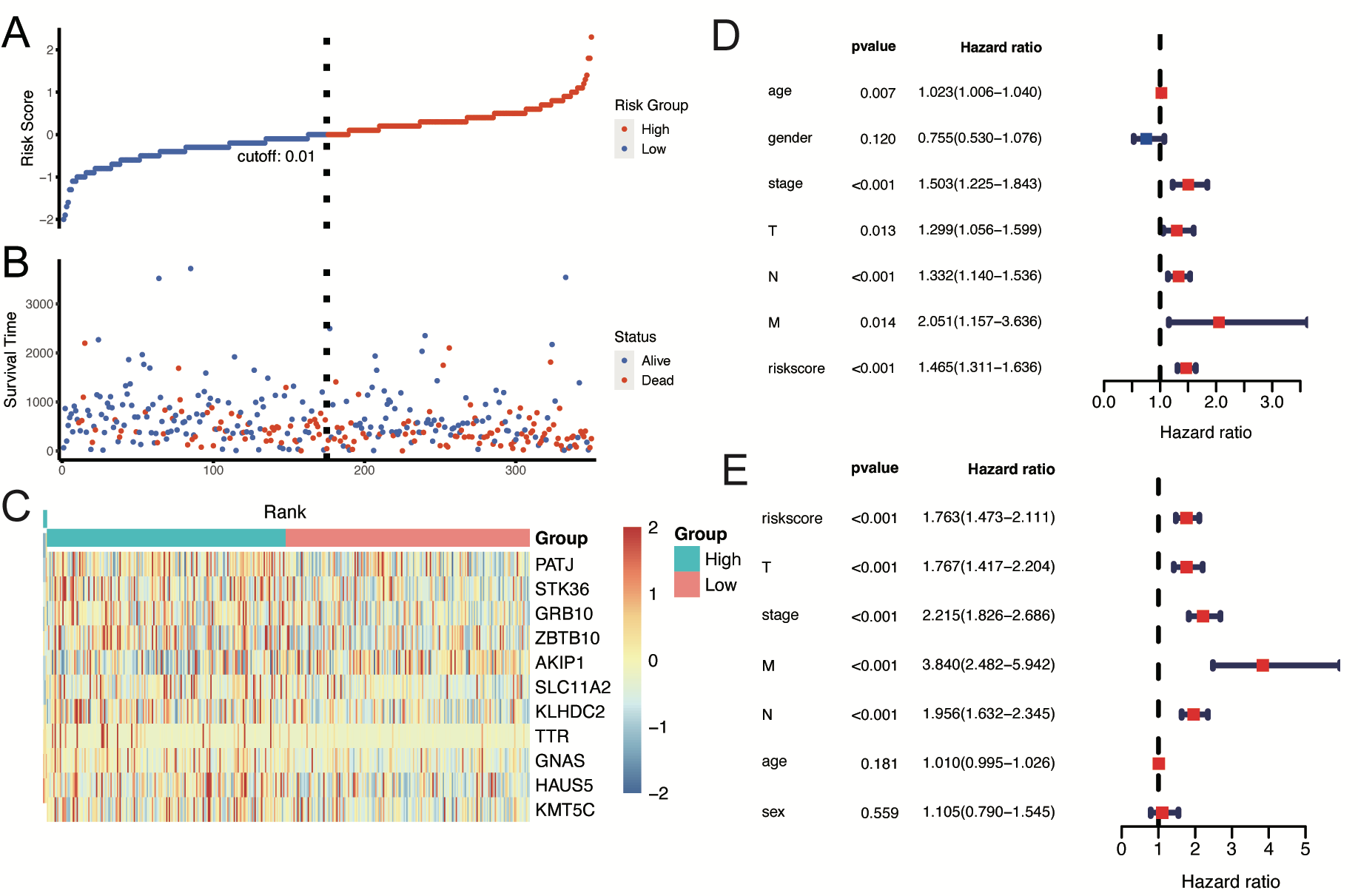


Figure S4. Establish a PRDM14-differential genomic and clinical prediction model for GC prognosis. (A) Distribution of high- and low-riskscore subgroups with the mean value (vertical dashed line). (B) Distribution of survival status in two riskscore subgroups. (C) Heatmap for the expression of genes in GC subgroups. (D)Univariate cox regression models are conducted to uncover the association of clinical features and riskscore with GC survival outcome in TCGA. (E)Univariate cox regression models to reveal the correlation between clinical characteristics and riskscore in GSE62254.


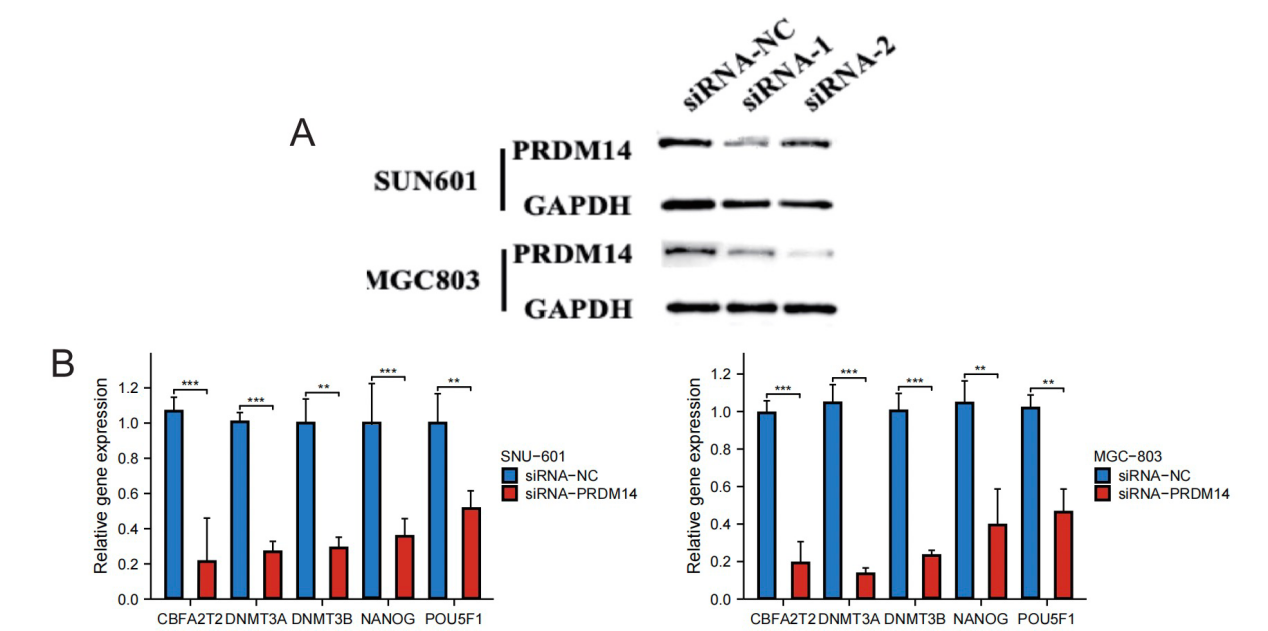


Figure S5. Western Blotting verified the expression of PRDM14 after treatment with two siRNAs.


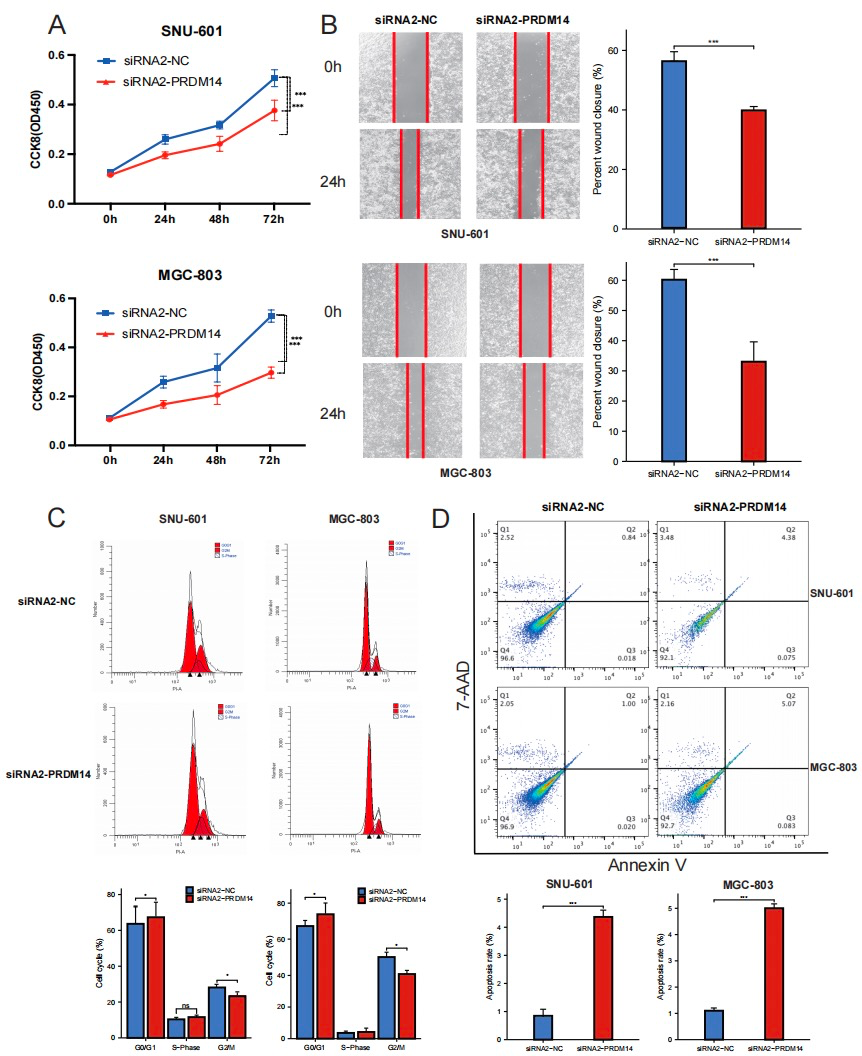


Figure S6. Silencing PRDM14 inhibits proliferation, migration, cell cycle, and promotes apoptosis with siRNA2. (A) Cell proliferation analysis with siRNA2-PRDM14 cell activity at different times (0 h, 24 h, 48 h, and 72 h). (B) Cell migration of SNU-601 and MGC-803 cells after siRNA2 treatment. (C) Cell cycle distribution of SNU-601 and MGC-803 cells after siRNA2 application. (D) Apoptosis of SNU-601 and MGC-803 cells at various stages after siRNA2 treatment.


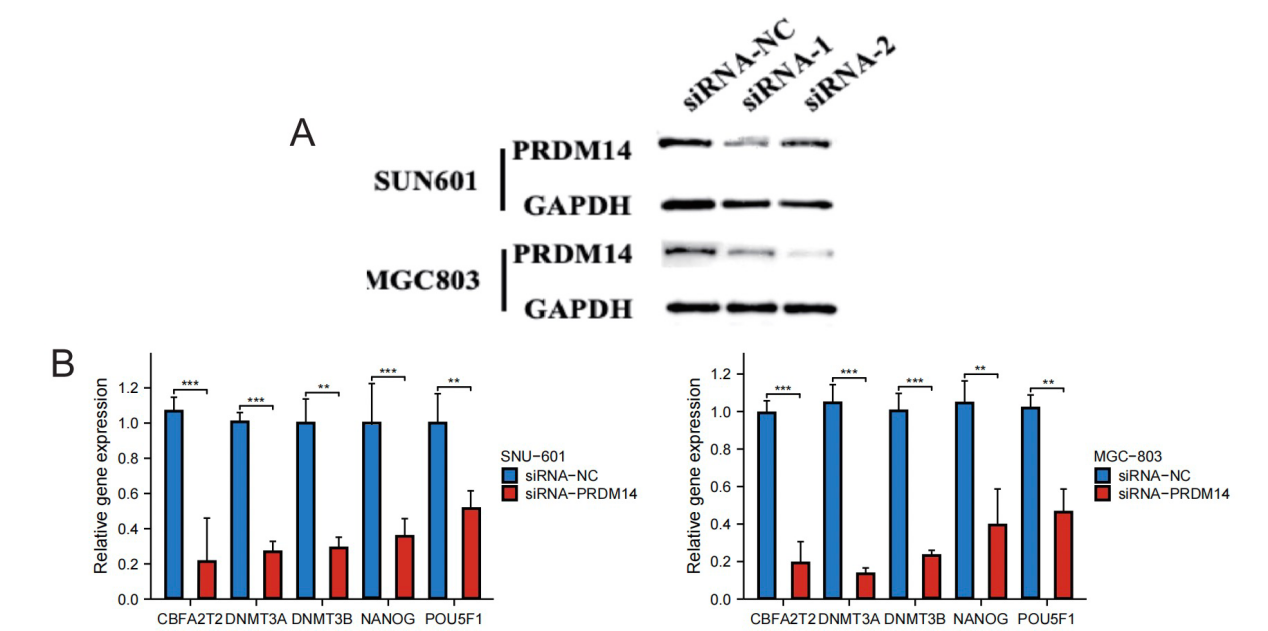


Figure S7. si2-PRDM14-mediated expression of associated regulatory genes.


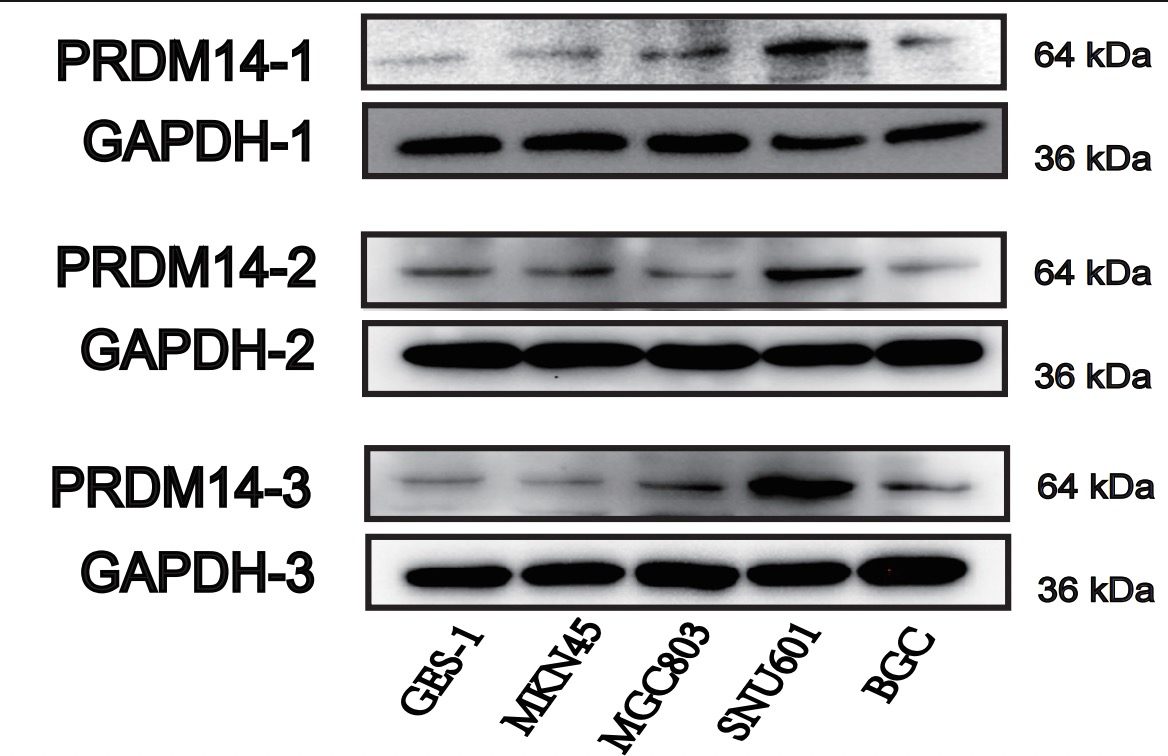


Figure S8. Western blotting analysis of PRDM14 expression in three GC cell lines, and three independent experiments were performed.

**Supplementary Tables**

Table S1 Primers of PRDM14 regulates potentially functionally relevant genes

| Gene | Forward | Reverse |
| --- | --- | --- |
| PRDM14 | TTTTATCGCCAAAGGAGT | CGGGCACAGTTGACATAG |
| GAPDH | ACAACTTTGGTATCGTGGAAGG | GCCATCACGCCACAGTTTC |
| CBFA2T2 | CCAGTGTCCTTCACTCCTACT | TTGCTGAGTTGTCGAGCACC |
| DNMT3A | CCTGCAATGACCTCTCCATT | CGGCCAGTACCCTCATAAAG |
| DNMT3B | AGGGAAGACTCGATCCTCGTC | GTGTGTAGCTTAGCAGACTGG |
| NANOG | TTTGTGGGCCTGAAGAAAACT | AGGGCTGTCCTGAATAAGCAG |
| POU5F1 | GTGTTCAGCCAAAAGACCATCT | GGCCTGCATGAGGGTTTCT |
| SOX2 | GCCGAGTGGAAACTTTTGTCG | GGCAGCGTGTACTTATCCTTCT |

Table S2 List of 146 PRDM4-related differential genes

| Number | GeneID | logFC | AveExpr | t | P.Value | adj.P.Val | B |
| --- | --- | --- | --- | --- | --- | --- | --- |
| 1 | SALL4 | 1.9890 | 2.1097 | 5.1715 | 0.0000 | 0.0001 | 6.2105 |
| 2 | KANSL2 | 0.9456 | 6.0555 | 4.5432 | 0.0000 | 0.0005 | 3.4438 |
| 3 | POU2F1 | 0.8016 | 4.2381 | 4.2182 | 0.0000 | 0.0013 | 2.1378 |
| 4 | CSPP1 | 0.8055 | 3.5022 | 4.2074 | 0.0000 | 0.0013 | 2.0961 |
| 5 | MOCS3 | 0.6548 | 2.9164 | 4.1455 | 0.0000 | 0.0016 | 1.8577 |
| 6 | ACVR1B | 3.3963 | 14.7218 | 4.0791 | 0.0001 | 0.0020 | 1.6058 |
| 7 | CHD7 | 0.9566 | 3.7713 | 4.0330 | 0.0001 | 0.0023 | 1.4328 |
| 8 | KMT2D | 1.5487 | 8.7737 | 3.8592 | 0.0001 | 0.0039 | 0.7978 |
| 9 | ZNF552 | 0.6471 | 3.0475 | 3.8572 | 0.0001 | 0.0039 | 0.7905 |
| 10 | ATP9A | 5.0005 | 20.7360 | 3.8364 | 0.0001 | 0.0042 | 0.7162 |
| 11 | CHD6 | 1.0320 | 4.9055 | 3.8184 | 0.0002 | 0.0044 | 0.6523 |
| 12 | BAZ2A | 1.8198 | 13.9686 | 3.8048 | 0.0002 | 0.0046 | 0.6040 |
| 13 | SLC43A2 | 1.2274 | 4.9808 | 3.8044 | 0.0002 | 0.0046 | 0.6026 |
| 14 | SYNE4 | 0.8636 | 1.8415 | 3.7718 | 0.0002 | 0.0050 | 0.4880 |
| 15 | HKR1 | 0.6215 | 3.4472 | 3.7430 | 0.0002 | 0.0054 | 0.3873 |
| 16 | SMARCD1 | 2.0743 | 14.8055 | 3.7300 | 0.0002 | 0.0056 | 0.3420 |
| 17 | PIGZ | 0.9626 | 2.6020 | 3.7264 | 0.0002 | 0.0057 | 0.3297 |
| 18 | ZSWIM1 | 1.4639 | 8.8623 | 3.7186 | 0.0002 | 0.0058 | 0.3026 |
| 19 | PAN2 | 0.8246 | 4.5031 | 3.7084 | 0.0002 | 0.0059 | 0.2675 |
| 20 | ZNF335 | 0.9242 | 5.6051 | 3.6872 | 0.0003 | 0.0063 | 0.1945 |
| 21 | ARID3A | 3.5888 | 5.7275 | 3.6702 | 0.0003 | 0.0066 | 0.1361 |
| 22 | ZNF764 | 0.7855 | 4.0149 | 3.6458 | 0.0003 | 0.0070 | 0.0530 |
| 23 | ADNP | 2.9835 | 19.7576 | 3.6435 | 0.0003 | 0.0070 | 0.0454 |
| 24 | ZMYND8 | 3.7838 | 15.3357 | 3.6358 | 0.0003 | 0.0072 | 0.0191 |
| 25 | UCKL1 | 2.3515 | 15.1613 | 3.6237 | 0.0003 | 0.0074 | -0.0218 |
| 26 | AKIP1 | -0.6079 | 5.4975 | -3.6053 | 0.0004 | 0.0078 | -0.0838 |
| 27 | AKAP8L | 0.6031 | 5.3899 | 3.5869 | 0.0004 | 0.0082 | -0.1456 |
| 28 | GSPT2 | 0.7741 | 2.0200 | 3.5656 | 0.0004 | 0.0088 | -0.2167 |
| 29 | PLXNB1 | 2.6210 | 12.1726 | 3.5317 | 0.0005 | 0.0096 | -0.3292 |
| 30 | ARFGAP1 | 1.9503 | 12.2517 | 3.5206 | 0.0005 | 0.0099 | -0.3656 |
| 31 | ARID1A | 2.5665 | 19.3985 | 3.5084 | 0.0005 | 0.0102 | -0.4056 |
| 32 | DNMT3A | 0.6250 | 3.0195 | 3.5072 | 0.0005 | 0.0103 | -0.4095 |
| 33 | HPN | 2.2810 | 2.8688 | 3.5059 | 0.0005 | 0.0103 | -0.4139 |
| 34 | ZNF587 | 0.8992 | 5.2141 | 3.5005 | 0.0005 | 0.0104 | -0.4314 |
| 35 | MDN1 | 0.6629 | 3.7686 | 3.4980 | 0.0005 | 0.0105 | -0.4395 |
| 36 | WIPF3 | 0.9565 | 1.9620 | 3.4917 | 0.0005 | 0.0107 | -0.4604 |
| 37 | ASXL1 | 1.8071 | 9.9282 | 3.4743 | 0.0006 | 0.0111 | -0.5168 |
| 38 | TRIM24 | 1.7846 | 7.4367 | 3.4546 | 0.0006 | 0.0117 | -0.5807 |
| 39 | PARP6 | 0.9223 | 3.6656 | 3.4523 | 0.0006 | 0.0118 | -0.5882 |
| 40 | TUBGCP6 | 0.8207 | 5.3784 | 3.4508 | 0.0006 | 0.0118 | -0.5930 |
| 41 | UQCC1 | 0.8000 | 5.1937 | 3.4432 | 0.0006 | 0.0120 | -0.6175 |
| 42 | CARD11 | 2.7376 | 7.2174 | 3.4362 | 0.0007 | 0.0123 | -0.6401 |
| 43 | ZNF629 | 0.9894 | 6.6872 | 3.4187 | 0.0007 | 0.0129 | -0.6961 |
| 44 | KIAA0355 | 0.8156 | 5.1311 | 3.3984 | 0.0008 | 0.0137 | -0.7608 |
| 45 | DDX27 | 2.7505 | 15.5583 | 3.3923 | 0.0008 | 0.0139 | -0.7803 |
| 46 | SOAT2 | 0.9646 | 0.7461 | 3.3879 | 0.0008 | 0.0140 | -0.7942 |
| 47 | CYTH2 | 0.6342 | 5.5304 | 3.3855 | 0.0008 | 0.0140 | -0.8019 |
| 48 | BRD8 | 0.7384 | 5.8161 | 3.3837 | 0.0008 | 0.0141 | -0.8075 |
| 49 | RNF114 | 3.0832 | 21.9306 | 3.3769 | 0.0008 | 0.0143 | -0.8291 |
| 50 | TP53INP1 | 1.4329 | 6.5511 | 3.3767 | 0.0008 | 0.0143 | -0.8297 |
| 51 | PCMTD2 | 2.1562 | 9.1850 | 3.3640 | 0.0008 | 0.0148 | -0.8698 |
| 52 | SENP5 | 0.6355 | 4.9485 | 3.3635 | 0.0008 | 0.0149 | -0.8713 |
| 53 | MZF1 | 0.6049 | 3.7481 | 3.3621 | 0.0009 | 0.0149 | -0.8757 |
| 54 | GDPD5 | 1.1320 | 2.5546 | 3.3566 | 0.0009 | 0.0151 | -0.8931 |
| 55 | GRB10 | 1.0929 | 5.0862 | 3.3463 | 0.0009 | 0.0155 | -0.9253 |
| 56 | HMOX2 | -1.5569 | 11.0001 | -3.3405 | 0.0009 | 0.0157 | -0.9435 |
| 57 | BCL2A1 | -2.7893 | 7.8071 | -3.3191 | 0.0010 | 0.0167 | -1.0104 |
| 58 | BRD1 | 1.1432 | 8.5446 | 3.3123 | 0.0010 | 0.0170 | -1.0316 |
| 59 | STK36 | 0.6290 | 3.7421 | 3.3033 | 0.0010 | 0.0173 | -1.0595 |
| 60 | NCOA3 | 2.0722 | 12.1209 | 3.2822 | 0.0011 | 0.0184 | -1.1246 |
| 61 | PMVK | -3.2982 | 24.1022 | -3.2814 | 0.0011 | 0.0184 | -1.1272 |
| 62 | POLE | 0.6784 | 4.1625 | 3.2782 | 0.0011 | 0.0186 | -1.1369 |
| 63 | GNAS | 19.6913 | 84.2051 | 3.2707 | 0.0012 | 0.0189 | -1.1599 |
| 64 | ZBTB10 | 0.6559 | 2.5051 | 3.2570 | 0.0012 | 0.0195 | -1.2021 |
| 65 | GATC | 0.7543 | 6.4429 | 3.2517 | 0.0013 | 0.0198 | -1.2182 |
| 66 | ERN1 | 0.5887 | 3.8331 | 3.2484 | 0.0013 | 0.0199 | -1.2282 |
| 67 | KMT2B | 1.3744 | 9.0740 | 3.2446 | 0.0013 | 0.0201 | -1.2399 |
| 68 | IGF2BP2 | 3.5451 | 14.6478 | 3.2285 | 0.0014 | 0.0211 | -1.2889 |
| 69 | SH3BGRL3 | -35.1049 | 197.8801 | -3.2056 | 0.0015 | 0.0224 | -1.3580 |
| 70 | DDX23 | 2.2089 | 20.6152 | 3.1968 | 0.0015 | 0.0228 | -1.3843 |
| 71 | KAT14 | 0.9226 | 5.8776 | 3.1962 | 0.0015 | 0.0229 | -1.3863 |
| 72 | ZBED4 | 0.8595 | 6.4291 | 3.1903 | 0.0015 | 0.0231 | -1.4040 |
| 73 | ADAM15 | -4.3502 | 27.7771 | -3.1854 | 0.0016 | 0.0234 | -1.4186 |
| 74 | COX19 | 0.6175 | 3.9567 | 3.1781 | 0.0016 | 0.0238 | -1.4403 |
| 75 | CAPZB | -8.6427 | 86.4067 | -3.1780 | 0.0016 | 0.0238 | -1.4409 |
| 76 | PCDHB5 | 0.6903 | 1.0094 | 3.1757 | 0.0016 | 0.0239 | -1.4477 |
| 77 | SVIP | -1.1554 | 7.5129 | -3.1674 | 0.0017 | 0.0243 | -1.4723 |
| 78 | ETNK2 | 0.8328 | 1.3872 | 3.1634 | 0.0017 | 0.0246 | -1.4841 |
| 79 | IGF2BP1 | 1.9037 | 1.9168 | 3.1489 | 0.0018 | 0.0255 | -1.5274 |
| 80 | TTR | 6.9961 | 5.2226 | 3.1482 | 0.0018 | 0.0255 | -1.5293 |
| 81 | PPM1H | 1.9572 | 5.8149 | 3.1475 | 0.0018 | 0.0256 | -1.5314 |
| 82 | ANGEL1 | 0.8485 | 5.8664 | 3.1384 | 0.0018 | 0.0262 | -1.5583 |
| 83 | FRAS1 | 0.9134 | 1.7850 | 3.1348 | 0.0019 | 0.0265 | -1.5690 |
| 84 | CSAD | 0.6222 | 2.7042 | 3.1330 | 0.0019 | 0.0266 | -1.5744 |
| 85 | GGA3 | 0.7936 | 7.5149 | 3.1232 | 0.0019 | 0.0273 | -1.6031 |
| 86 | ZNF217 | 2.9277 | 13.5858 | 3.1207 | 0.0019 | 0.0274 | -1.6104 |
| 87 | NEO1 | 2.2709 | 12.3087 | 3.1138 | 0.0020 | 0.0279 | -1.6307 |
| 88 | HAUS5 | 0.6203 | 3.5656 | 3.1101 | 0.0020 | 0.0282 | -1.6417 |
| 89 | SETDB1 | 0.7344 | 5.9482 | 3.1088 | 0.0020 | 0.0282 | -1.6455 |
| 90 | INPP5E | 0.7083 | 5.3736 | 3.1085 | 0.0020 | 0.0282 | -1.6462 |
| 91 | LAMA5 | 5.2201 | 21.4206 | 3.1025 | 0.0021 | 0.0286 | -1.6637 |
| 92 | EDC3 | 0.6891 | 5.4808 | 3.0940 | 0.0021 | 0.0293 | -1.6884 |
| 93 | AKR7A2 | -4.5986 | 28.9386 | -3.0931 | 0.0021 | 0.0294 | -1.6912 |
| 94 | DPM1 | 5.7110 | 39.2110 | 3.0929 | 0.0021 | 0.0294 | -1.6917 |
| 95 | PLAGL2 | 4.8009 | 15.2465 | 3.0897 | 0.0022 | 0.0295 | -1.7011 |
| 96 | USP36 | 0.6968 | 6.2397 | 3.0852 | 0.0022 | 0.0298 | -1.7141 |
| 97 | KLHDC2 | 0.6404 | 4.9395 | 3.0845 | 0.0022 | 0.0298 | -1.7162 |
| 98 | DIDO1 | 1.1884 | 8.8256 | 3.0705 | 0.0023 | 0.0308 | -1.7566 |
| 99 | CSRNP2 | 0.6417 | 6.3709 | 3.0638 | 0.0023 | 0.0313 | -1.7761 |
| 100 | ERBB3 | 7.7824 | 40.4057 | 3.0572 | 0.0024 | 0.0319 | -1.7951 |
| 101 | DNMT3B | 1.1153 | 2.4788 | 3.0512 | 0.0024 | 0.0323 | -1.8122 |
| 102 | DCP1A | 0.7331 | 8.8397 | 3.0512 | 0.0024 | 0.0323 | -1.8123 |
| 103 | ABCC4 | 1.1052 | 3.9648 | 3.0488 | 0.0025 | 0.0325 | -1.8192 |
| 104 | PPP6R2 | 1.1703 | 10.7188 | 3.0431 | 0.0025 | 0.0330 | -1.8356 |
| 105 | SLC25A36 | 1.0312 | 7.2508 | 3.0428 | 0.0025 | 0.0330 | -1.8365 |
| 106 | HADH | -2.7742 | 18.7195 | -3.0370 | 0.0026 | 0.0334 | -1.8531 |
| 107 | LRRN1 | 1.5963 | 2.1788 | 3.0367 | 0.0026 | 0.0334 | -1.8539 |
| 108 | SCAF4 | 0.9226 | 9.1909 | 3.0297 | 0.0026 | 0.0340 | -1.8740 |
| 109 | LGR5 | 3.4813 | 6.1908 | 3.0254 | 0.0027 | 0.0343 | -1.8862 |
| 110 | PHACTR4 | 1.2411 | 12.1845 | 3.0242 | 0.0027 | 0.0344 | -1.8895 |
| 111 | EPDR1 | 1.4054 | 4.8965 | 3.0222 | 0.0027 | 0.0345 | -1.8954 |
| 112 | BCAM | 9.1271 | 22.6501 | 3.0127 | 0.0028 | 0.0353 | -1.9222 |
| 113 | PDZK1 | 0.7438 | 1.4241 | 3.0127 | 0.0028 | 0.0353 | -1.9223 |
| 114 | SESN1 | 2.0896 | 7.3608 | 2.9971 | 0.0029 | 0.0365 | -1.9665 |
| 115 | TULP4 | 0.8518 | 4.6915 | 2.9866 | 0.0030 | 0.0373 | -1.9960 |
| 116 | TRIM44 | 1.6966 | 10.7801 | 2.9862 | 0.0030 | 0.0373 | -1.9973 |
| 117 | FADS2 | 1.7730 | 4.9576 | 2.9764 | 0.0031 | 0.0382 | -2.0249 |
| 118 | RALGAPB | 1.1409 | 8.4273 | 2.9420 | 0.0035 | 0.0413 | -2.1209 |
| 119 | UBAP2L | 2.4581 | 26.6262 | 2.9410 | 0.0035 | 0.0414 | -2.1237 |
| 120 | FADS1 | 0.8507 | 2.8723 | 2.9344 | 0.0035 | 0.0421 | -2.1419 |
| 121 | ATG4B | 0.5971 | 6.9940 | 2.9339 | 0.0036 | 0.0421 | -2.1434 |
| 122 | SLC11A2 | 1.2926 | 7.1710 | 2.9306 | 0.0036 | 0.0424 | -2.1523 |
| 123 | VPS4B | -1.8295 | 14.8688 | -2.9284 | 0.0036 | 0.0426 | -2.1585 |
| 124 | GBP3 | -3.7181 | 15.8429 | -2.9262 | 0.0036 | 0.0428 | -2.1647 |
| 125 | GUCA2A | 2.6311 | 2.9178 | 2.9257 | 0.0036 | 0.0428 | -2.1660 |
| 126 | BMF | 1.1497 | 4.9600 | 2.9209 | 0.0037 | 0.0432 | -2.1792 |
| 127 | PFAS | 0.7442 | 4.9166 | 2.9180 | 0.0037 | 0.0435 | -2.1872 |
| 128 | NINL | 0.7710 | 2.6315 | 2.9092 | 0.0038 | 0.0442 | -2.2116 |
| 129 | POFUT1 | 4.4577 | 19.2893 | 2.9068 | 0.0039 | 0.0445 | -2.2180 |
| 130 | TROAP | 1.2800 | 6.6943 | 2.8924 | 0.0040 | 0.0462 | -2.2575 |
| 131 | KANSL3 | 0.6396 | 6.7572 | 2.8842 | 0.0042 | 0.0470 | -2.2798 |
| 132 | URAD | 0.7216 | 0.4936 | 2.8826 | 0.0042 | 0.0472 | -2.2841 |
| 133 | USP24 | 0.8161 | 7.4087 | 2.8787 | 0.0042 | 0.0475 | -2.2947 |
| 134 | ZBTB12 | 0.8245 | 3.4299 | 2.8731 | 0.0043 | 0.0480 | -2.3100 |
| 135 | PATJ | 0.8620 | 6.4217 | 2.8711 | 0.0043 | 0.0482 | -2.3153 |
| 136 | RHOC | -4.7308 | 34.8189 | -2.8701 | 0.0043 | 0.0483 | -2.3182 |
| 137 | BCORL1 | 0.6419 | 3.8315 | 2.8696 | 0.0043 | 0.0483 | -2.3194 |
| 138 | NCOA5 | 1.6620 | 13.8148 | 2.8662 | 0.0044 | 0.0487 | -2.3286 |
| 139 | CELF1 | 0.9373 | 11.8312 | 2.8645 | 0.0044 | 0.0490 | -2.3334 |
| 140 | SIN3A | 0.6889 | 6.5286 | 2.8637 | 0.0044 | 0.0490 | -2.3354 |
| 141 | MORC2 | 0.7327 | 6.9358 | 2.8631 | 0.0044 | 0.0491 | -2.3370 |
| 142 | LMNA | -7.3654 | 62.8760 | -2.8628 | 0.0044 | 0.0491 | -2.3380 |
| 143 | NBL1 | -3.1323 | 17.6143 | -2.8592 | 0.0045 | 0.0495 | -2.3476 |
| 144 | HNF1B | 2.1956 | 6.5032 | 2.8591 | 0.0045 | 0.0495 | -2.3480 |
| 145 | KMT5C | 0.6019 | 3.5292 | 2.8574 | 0.0045 | 0.0497 | -2.3526 |
| 146 | ZDHHC8 | 1.1561 | 8.9738 | 2.8570 | 0.0045 | 0.0497 | -2.3536 |
