# Supplementary material for: Integrated analysis of the role of PR/SET domain 14 in gastric cancer
Source: BMC Cancer. 2024 Jun 5;24:685. doi: 10.1186/s12885-024-12424-1 (PMC11151633; doi:10.1186/s12885-024-12424-1)

**Western blotting of PRDM14**

**PRDM14-1:**


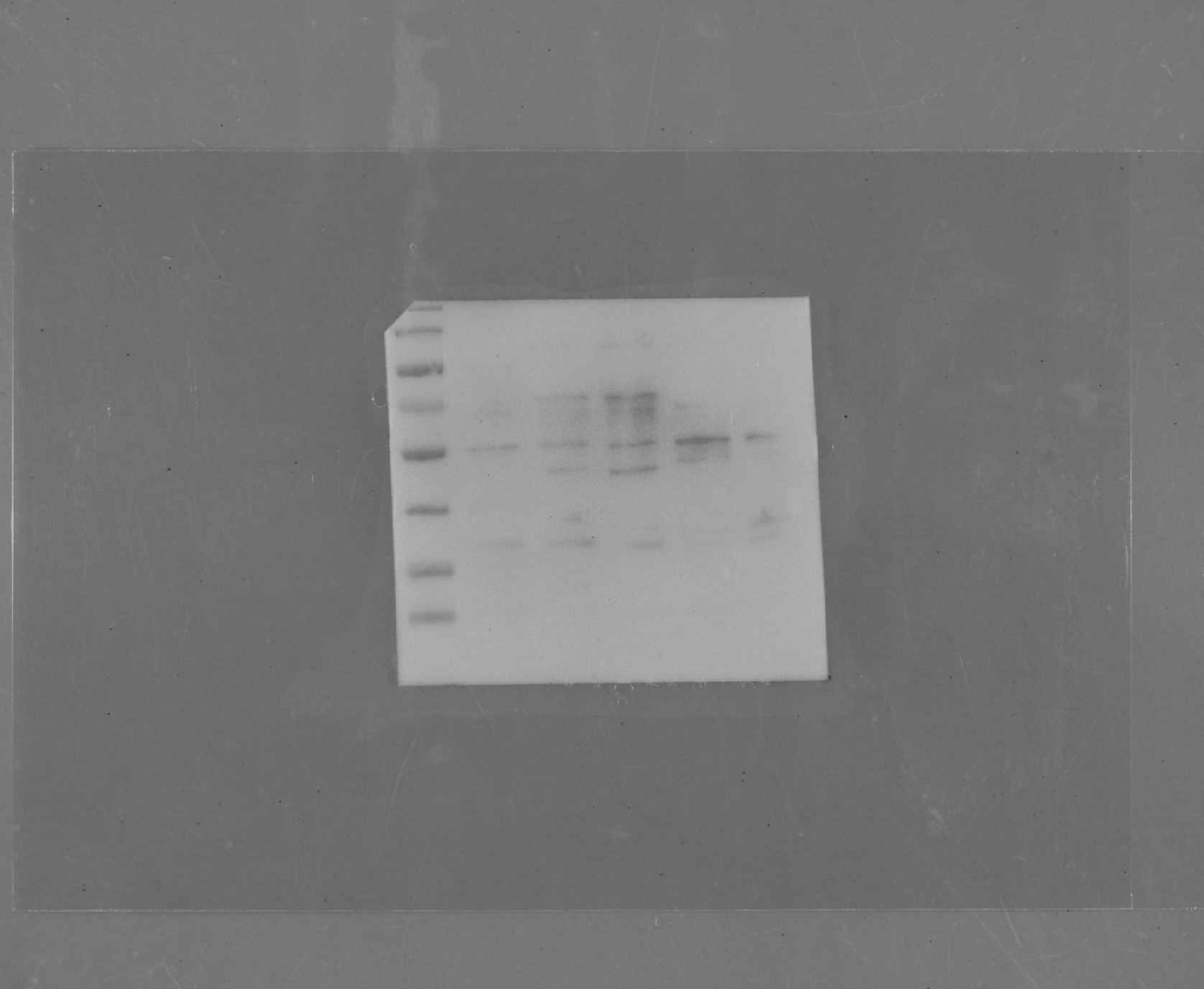


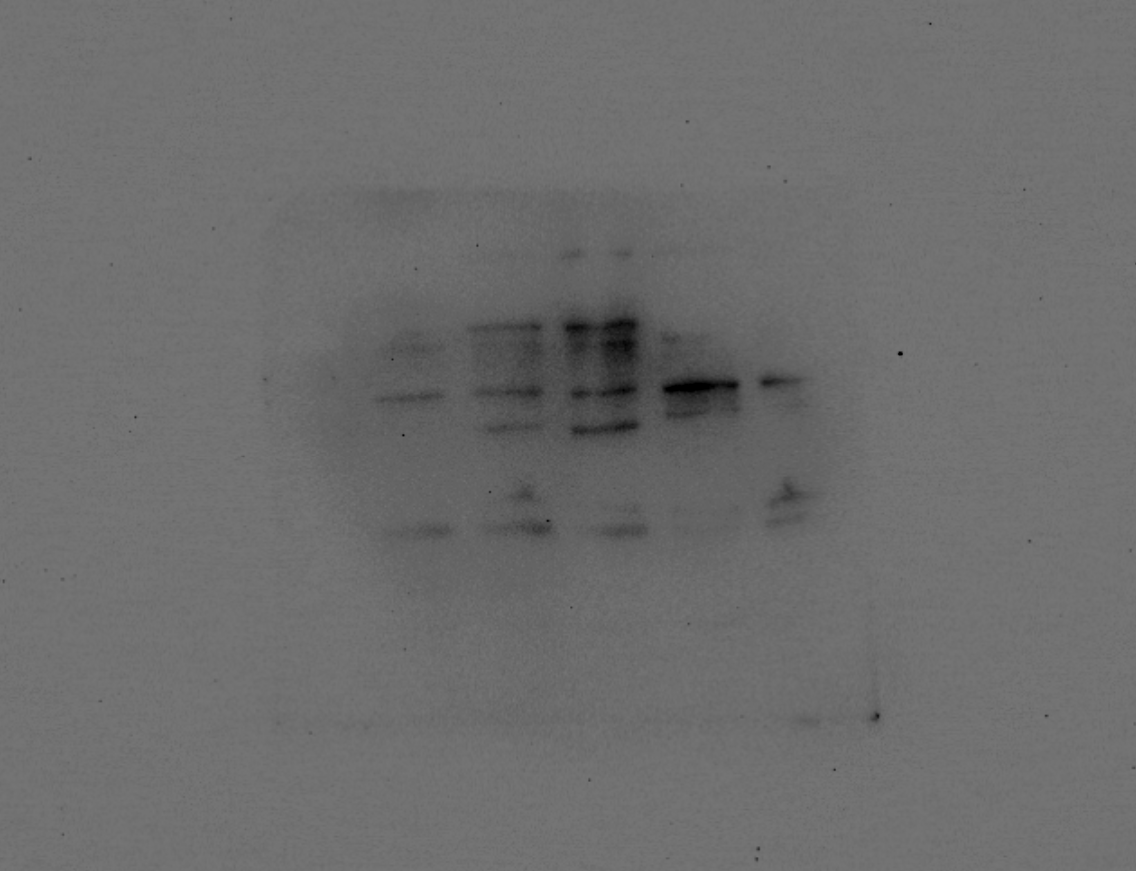


**GAPDH-1:**


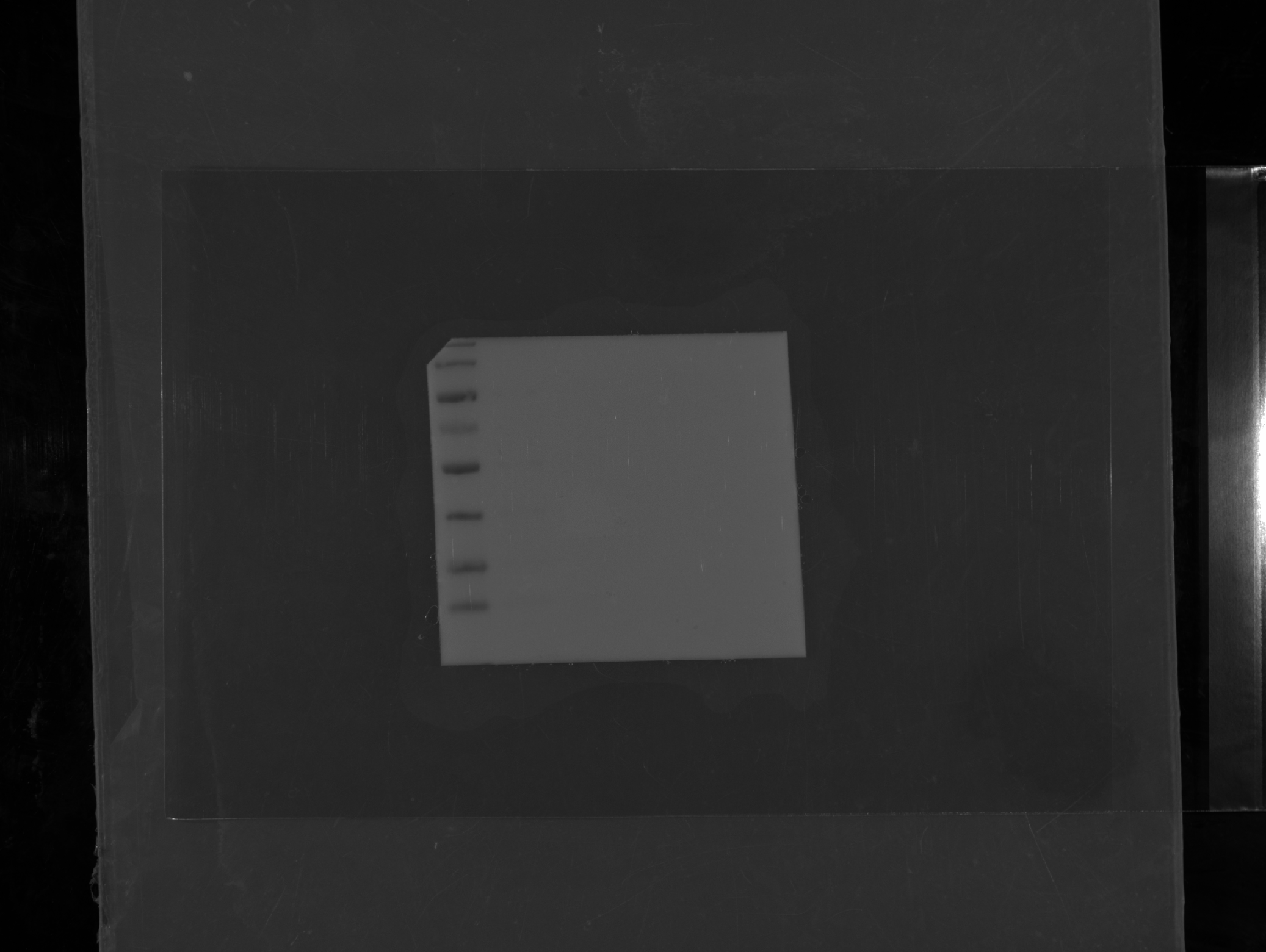


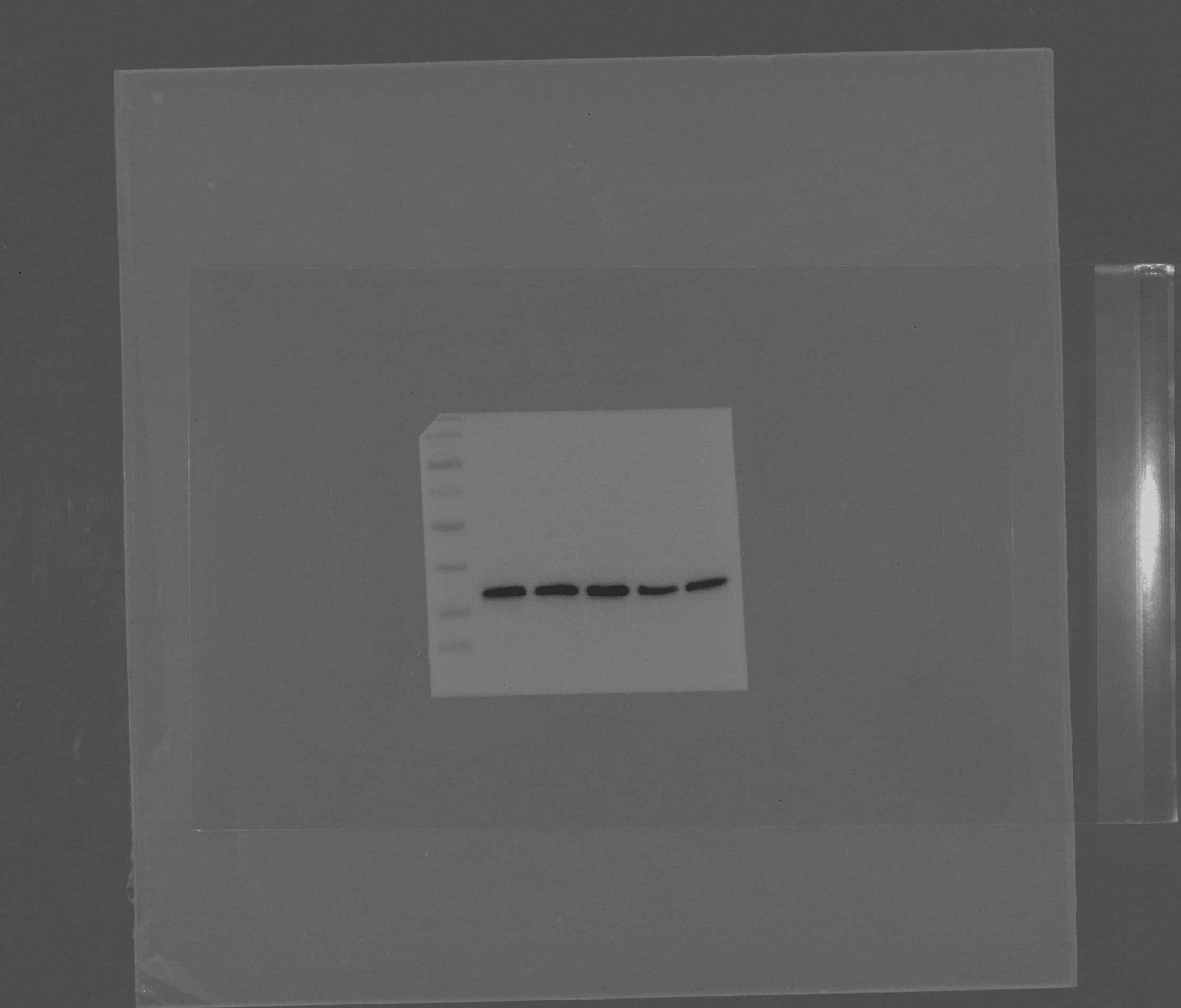


**PRDM14-2:**


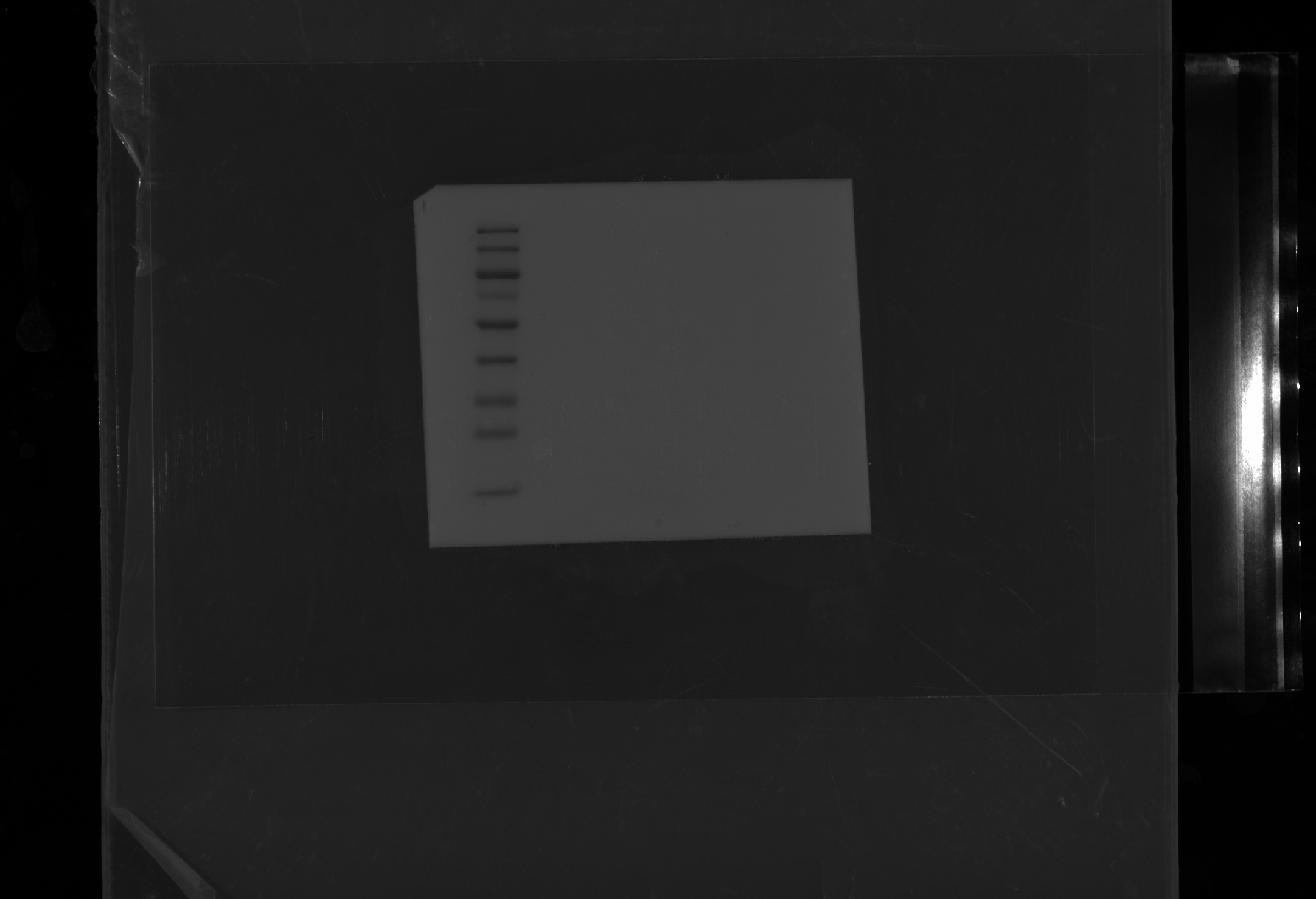


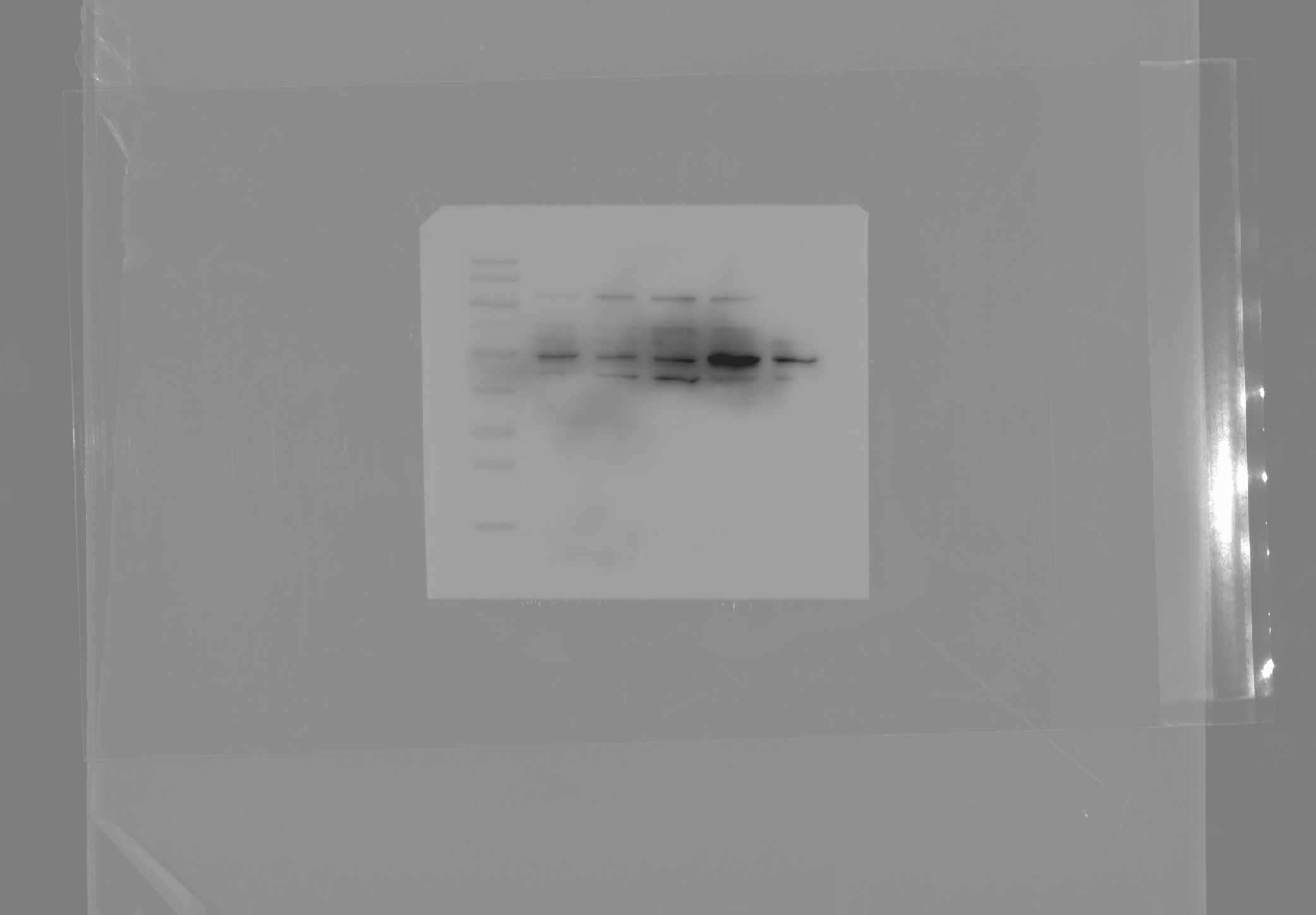


**GAPDH-2:**


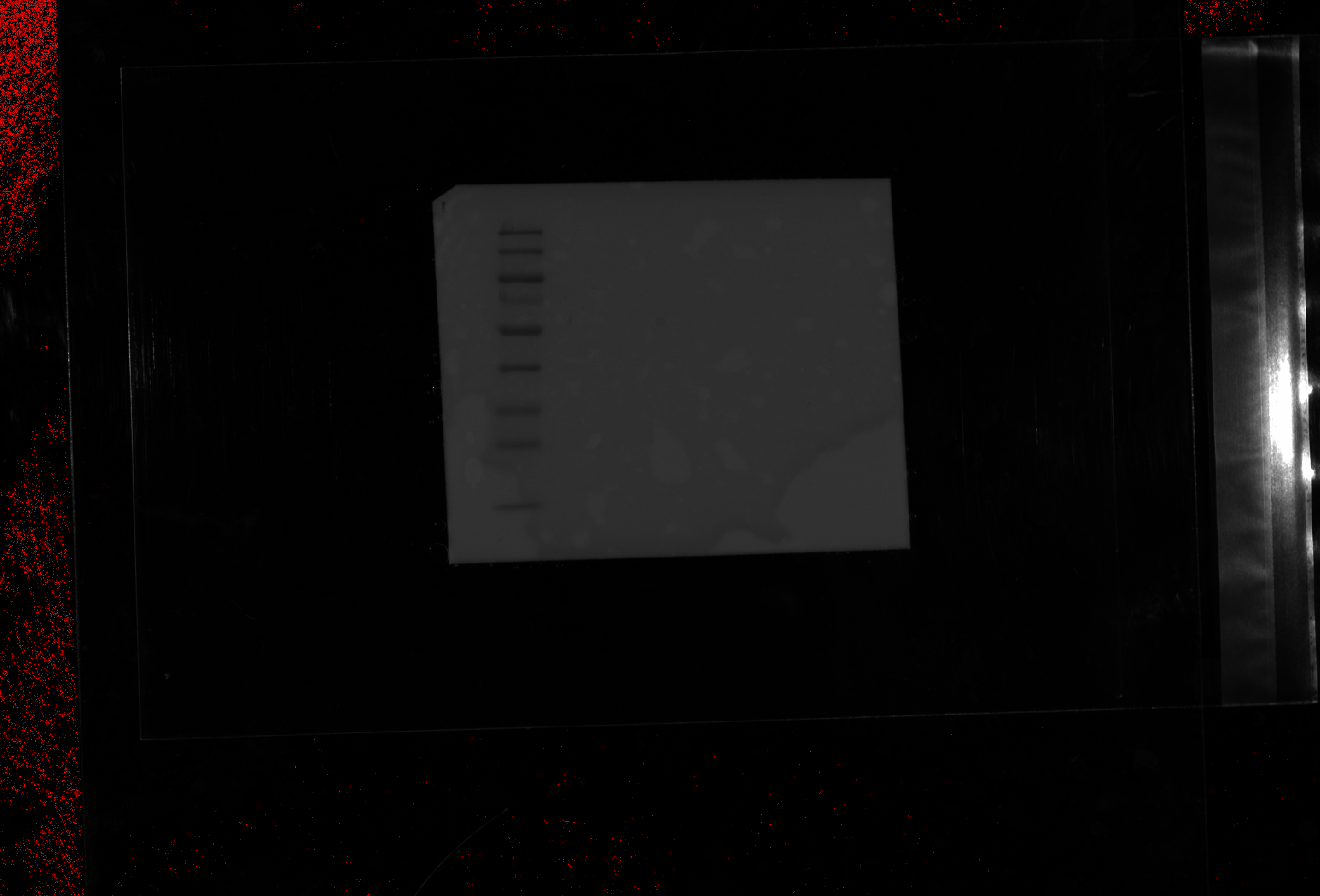


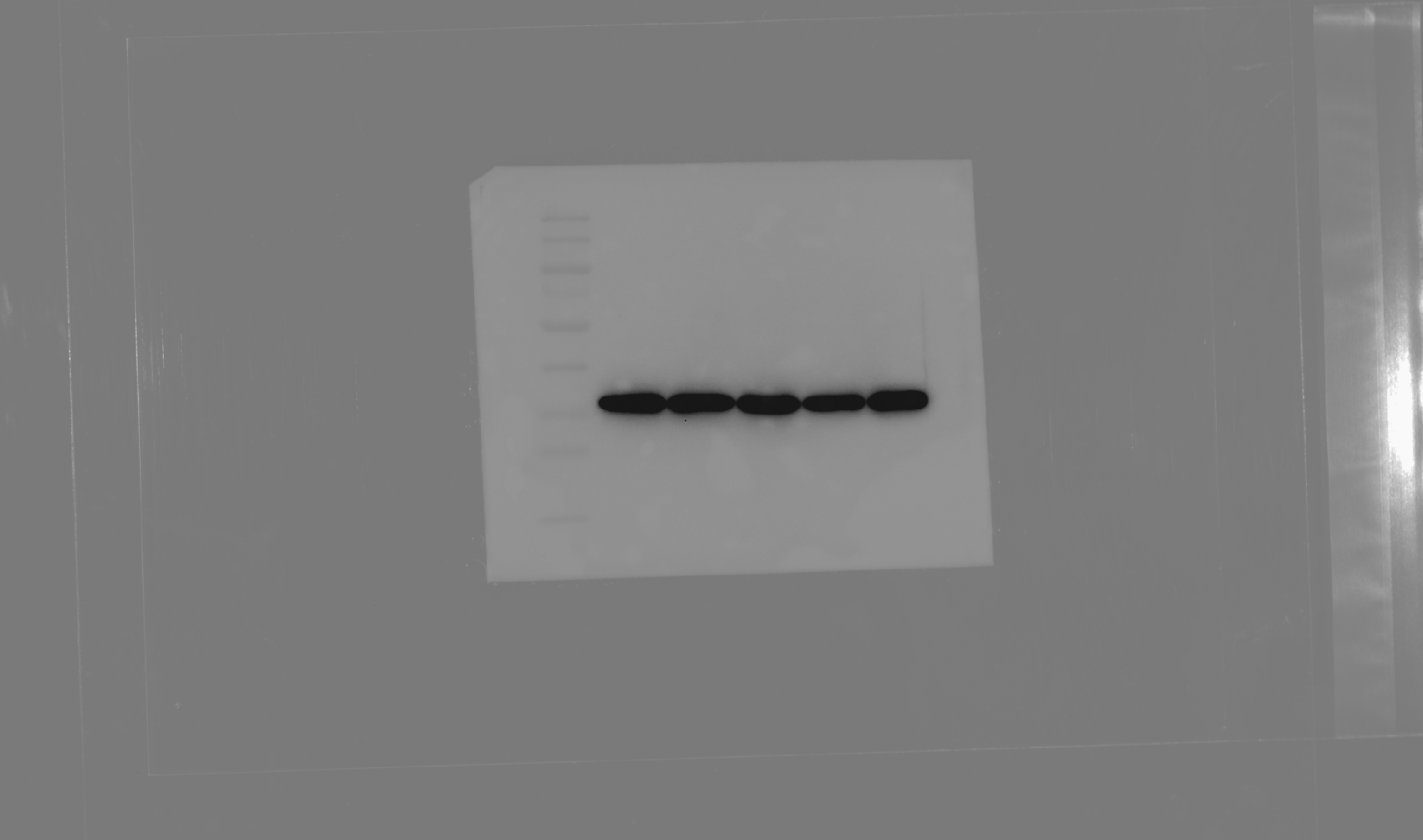


**PRDM14-3:**


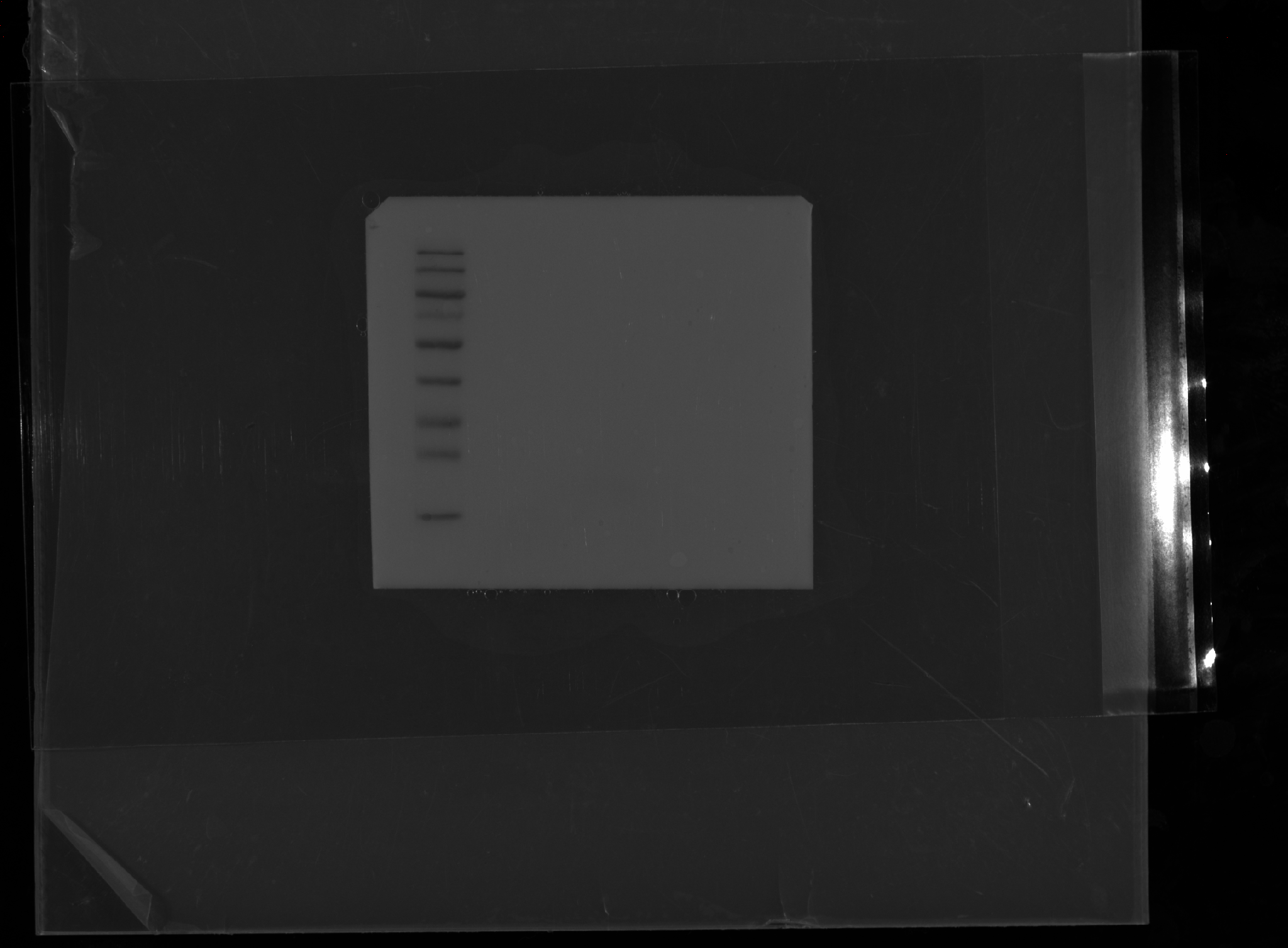


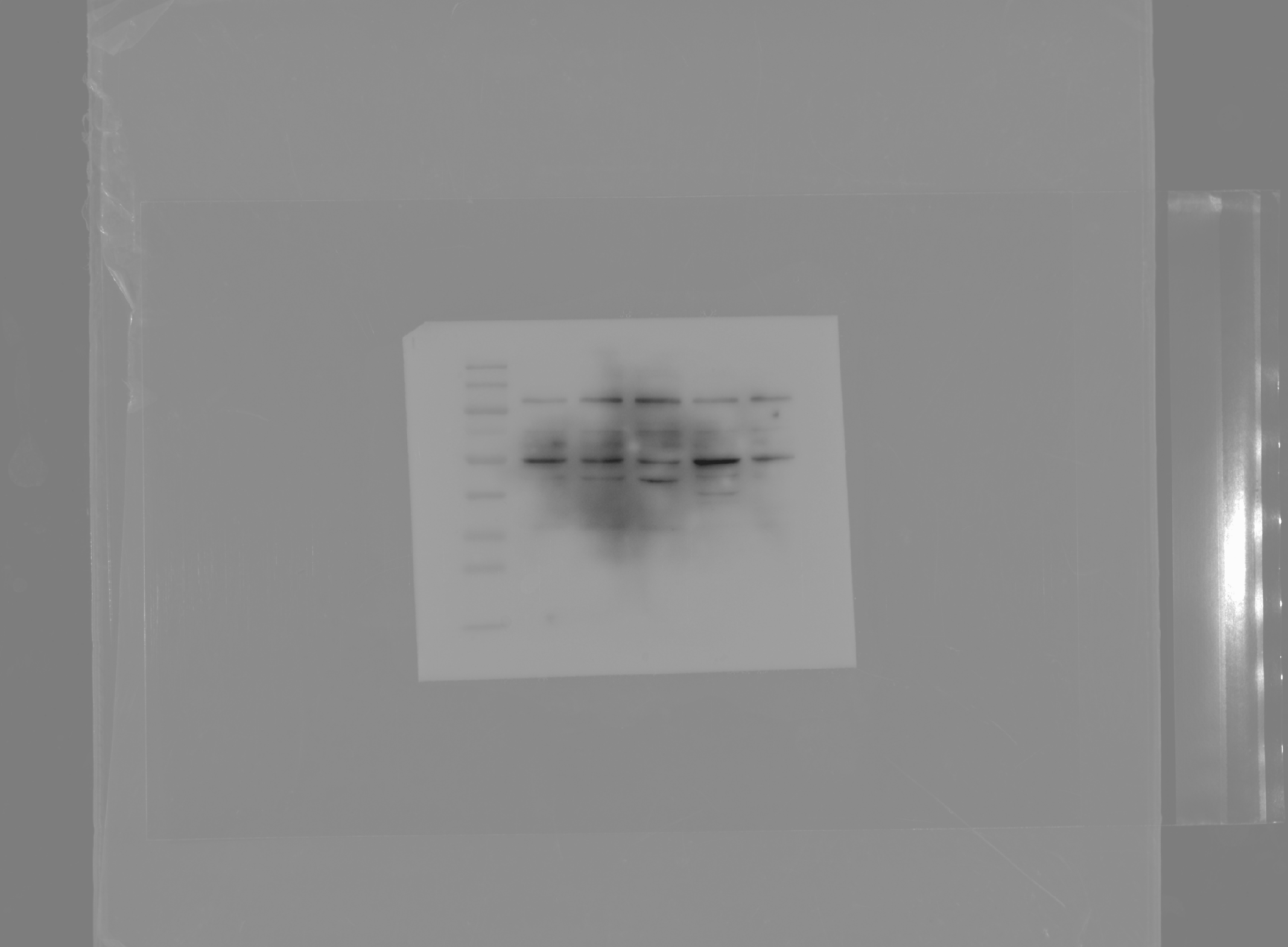


**GAPDH-3:**


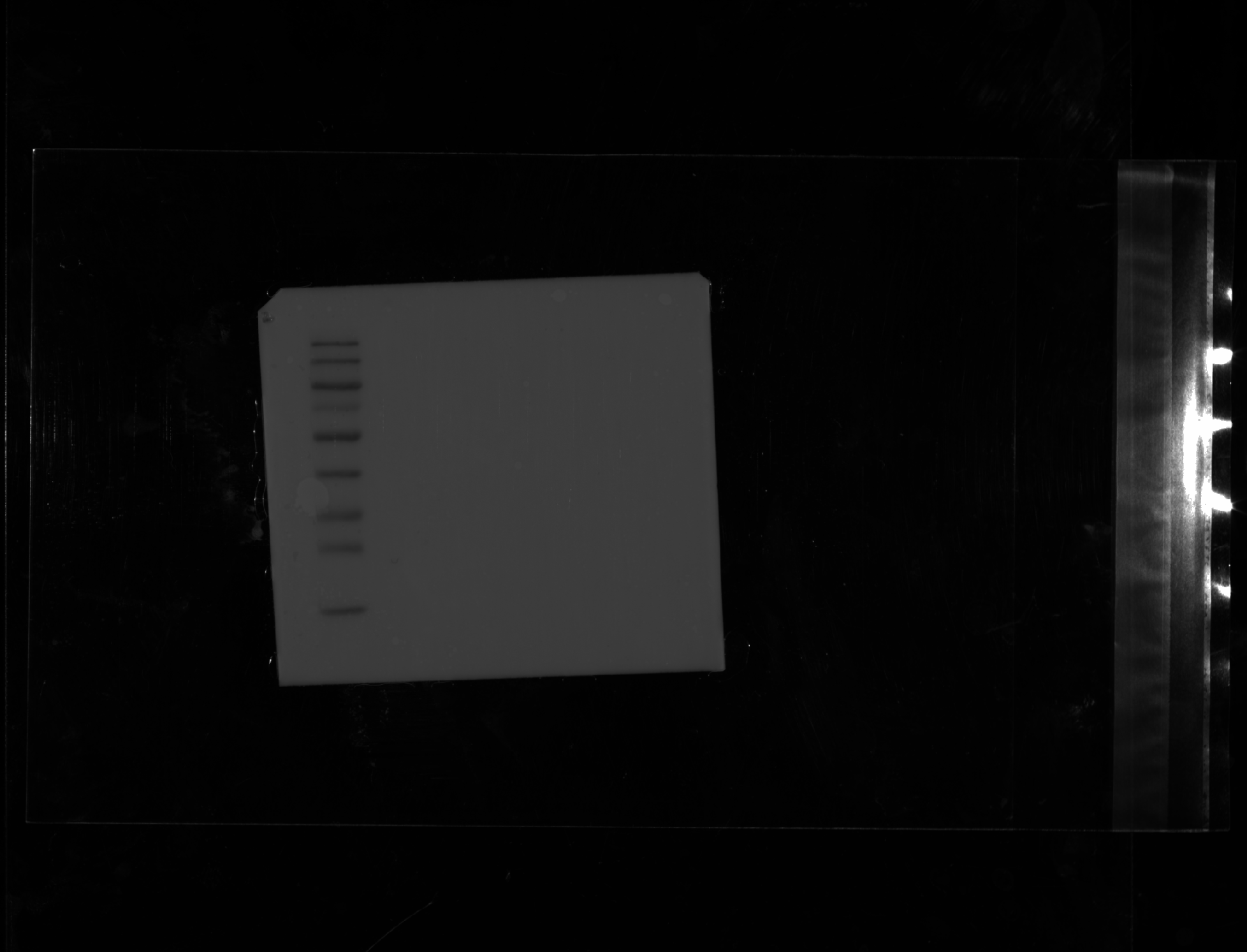


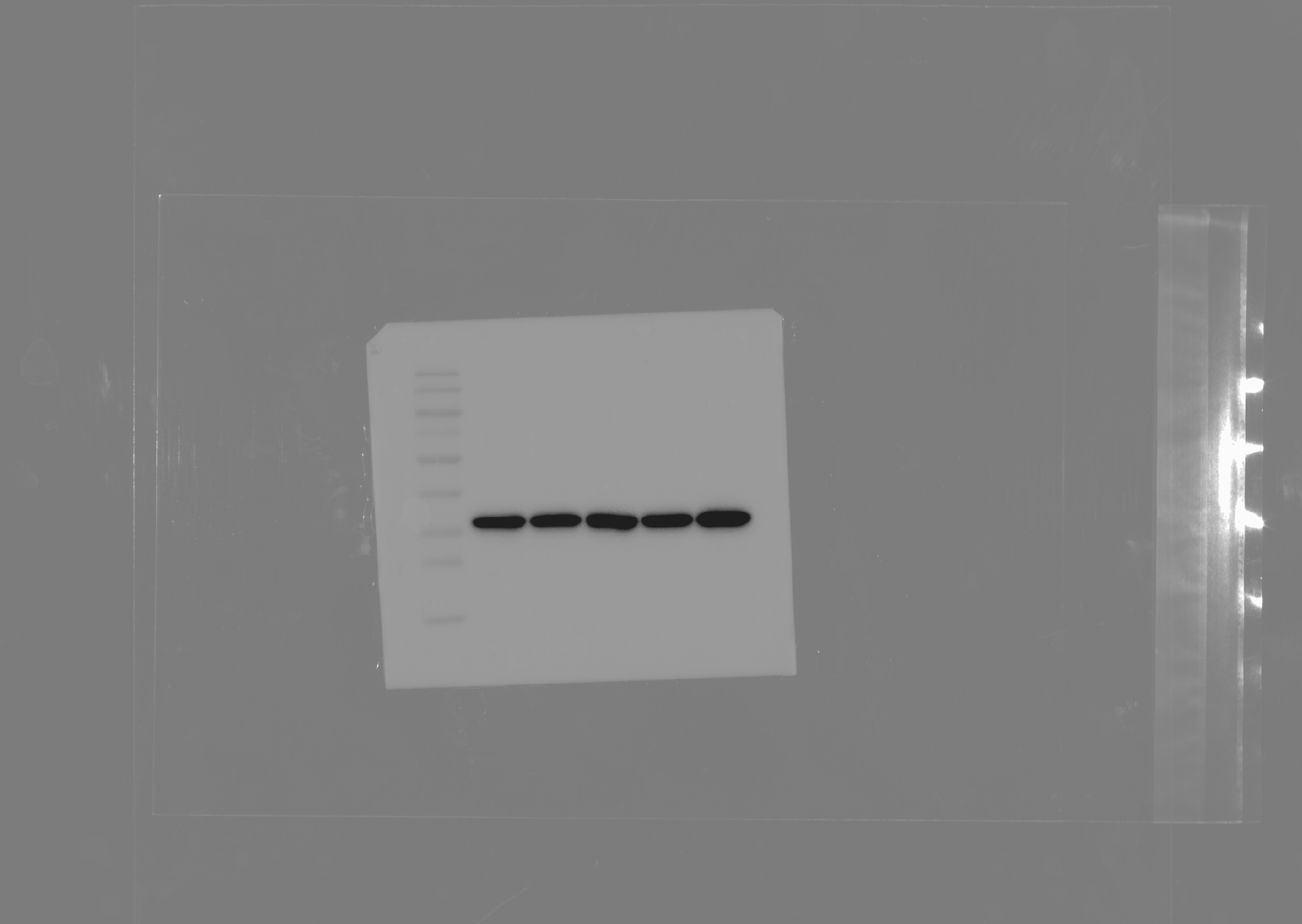


**Western Blot of PRDM14-siRNAs**

Our blots of PRDM14-siRNAs were cut before the antibody was attached, but we can ensure the authenticity of the data.

**PRDM14:**


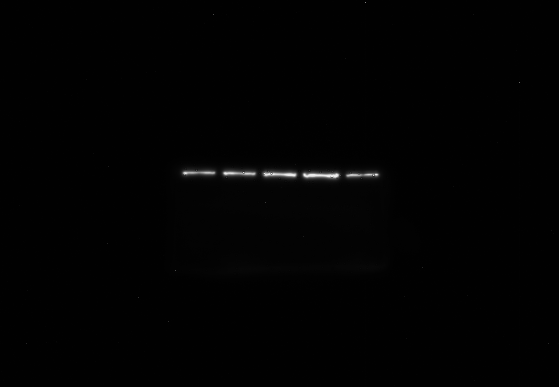


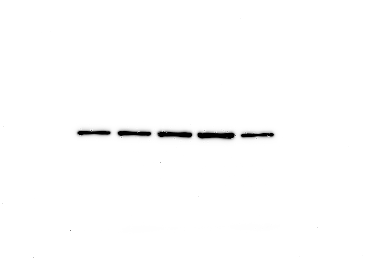


**PRDM14-siRNAs:**


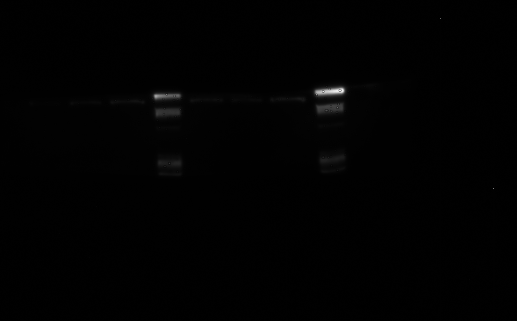


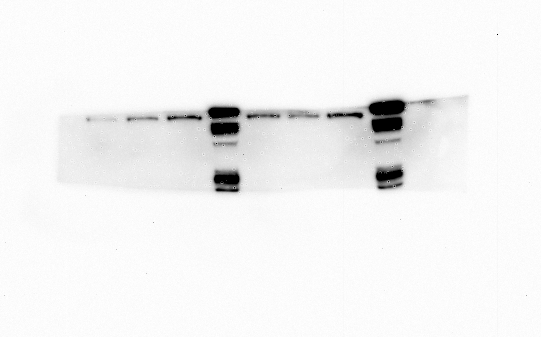


**PRDM14-GAPDH:**


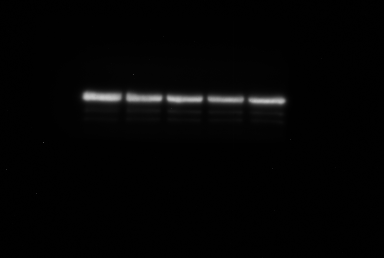


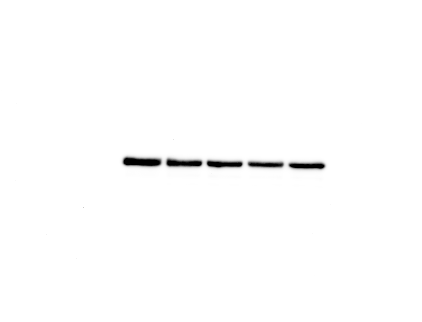


**PRDM14-siRNAs GAPDH:**


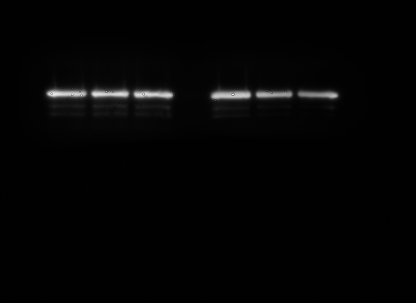


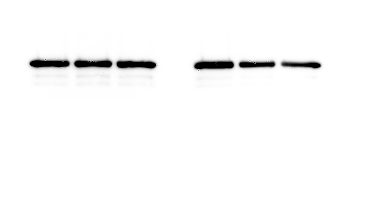

Supplement: Supplementary file 2 — Supplementary Material 2 [file 12885_2024_12424_MOESM2_ESM.docx]
